# Comparison of statistical methods used to meta-analyse results from interrupted time series studies: an empirical study

**DOI:** 10.1186/s12874-024-02147-z

**Published:** 2024-02-10

**Authors:** Elizabeth Korevaar, Simon L. Turner, Andrew B. Forbes, Amalia Karahalios, Monica Taljaard, Joanne E. McKenzie

**Affiliations:** 1 School of Public Health and Preventive Medicine, Monash University, Melbourne, VIC 3004 Australia; 2 Centre for Epidemiology and Biostatistics, Melbourne School of Population and Global Health, University of Melbourne, Melbourne, VIC 3010 Australia; 3 Clinical Epidemiology Program, Ottawa Hospital Research Institute, Ottawa, ON K1Y 4E9 Canada; 4 School of Epidemiology and Public Health, University of Ottawa, Ottawa, ON K1N 6N5 Canada

**Keywords:** Meta-analysis, Interrupted time series, Segmented regression, Statistical methods, Empirical study

## Abstract

**Background:**

The Interrupted Time Series (ITS) is a robust design for evaluating public health and policy interventions or exposures when randomisation may be infeasible. Several statistical methods are available for the analysis and meta-analysis of ITS studies. We sought to empirically compare available methods when applied to real-world ITS data.

**Methods:**

We sourced ITS data from published meta-analyses to create an online data repository. Each dataset was re-analysed using two ITS estimation methods. The level- and slope-change effect estimates (and standard errors) were calculated and combined using fixed-effect and four random-effects meta-analysis methods. We examined differences in meta-analytic level- and slope-change estimates, their 95% confidence intervals, p-values, and estimates of heterogeneity across the statistical methods.

**Results:**

Of 40 eligible meta-analyses, data from 17 meta-analyses including 282 ITS studies were obtained (predominantly investigating the effects of public health interruptions (88%)) and analysed. We found that on *average*, the meta-analytic effect estimates, their standard errors and between-study variances were not sensitive to meta-analysis method choice, irrespective of the ITS analysis method. However, across ITS analysis methods, for any given meta-analysis, there could be small to moderate differences in meta-analytic effect estimates, and important differences in the meta-analytic standard errors. Furthermore, the confidence interval widths and *p*-values for the meta-analytic effect estimates varied depending on the choice of confidence interval method and ITS analysis method.

**Conclusions:**

Our empirical study showed that meta-analysis effect estimates, their standard errors, confidence interval widths and *p*-values can be affected by statistical method choice. These differences may importantly impact interpretations and conclusions of a meta-analysis and suggest that the statistical methods are not interchangeable in practice.

**Supplementary Information:**

The online version contains supplementary material available at 10.1186/s12874-024-02147-z.

## Introduction

Systematic reviews may be used to collate and synthesise evidence on the effects of interventions targeted at populations (e.g., effects of a country-wide ban on smoking rates [1]) or the impacts of exposures (e.g., impacts of flooding events [2]). These reviews may include evidence beyond randomised trials by necessity, because trials may not be possible (in the case of exposures) or feasible (in the case of interventions targeted at populations) [3]. The interrupted time series (ITS) may be considered for inclusion in such reviews because this design is often used to examine population-level interventions and exposures, when randomisation is not possible (e.g., for ethical reasons, when a policy targets an entire population). Furthermore, this design is considered a robust alternative for evaluating the impact of population-level interventions / exposures [4–7]. The results across the included ITS studies may be statistically combined using meta-analysis, providing a combined estimate of the intervention / exposure’s impact [8, 9].

**Fig. 1:**
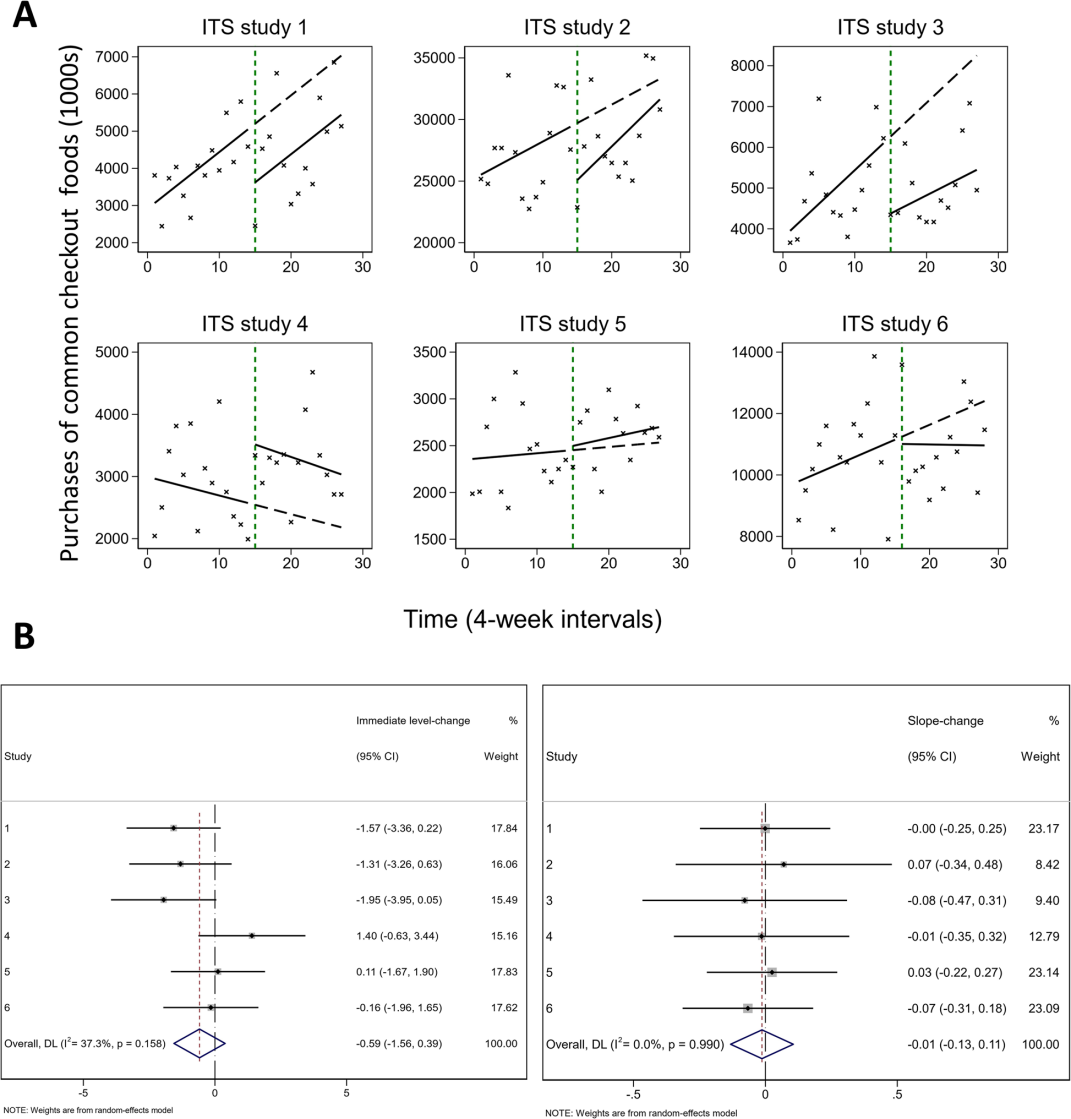
**A** Six interrupted time series (ITS) studies examining the effect of supermarket policies on purchases of common checkout foods [11]. The crosses represent data points, the solid lines represent the pre- and post-interruption trend lines and the dashed line represents the counterfactual trend line. The vertical dashed green line indicates the time of the interruption. **B** Forest plots depicting study-level and meta-analysis estimates of immediate level-change (left) and slope-change (right). *ITS* interrupted time series

In a classical ITS study, data are collected over time both before and after an intervention or exposure (henceforth referred to as an ‘interruption’), and aggregated using summary statistics over regular time intervals [10]. For example, in Ejlerskov et al. [11]*,* the interruptions examined were policies implemented in six supermarkets that aimed to reduce the purchasing of less-healthy foods that are commonly displayed at supermarket checkouts. The outcome examined was the number of checkout food purchases, aggregated into four-weekly periods (Fig. 1, Additional file 1: Figure S1) [11]. While the ITS design may also be used to examine the effects of an intervention on individuals (in which multiple measurements are taken before and after the intervention for each individual), we do not consider the use of the ITS design in this context further [12, 13].

In the analysis of data from this classical ITS design, a commonly fitted model structure is the segmented linear model [14, 15]. This model allows estimation of separate trends before and after the interruption (referred to as the pre- and post-interruption trends). Hence the advantage of the ITS design is that the series acts as its own control; the pre-interruption trend can be projected into the post-interruption period, which, when modelled correctly, provides a counterfactual for what would have occurred in the absence of the interruption [5, 14, 15]. The impact of the interruption can then be estimated by comparing the counterfactual with the observed post-interruption trend. A variety of effect metrics can be calculated, including level-change (e.g., immediately following the interruption) and slope-change [7, 16].

When estimating the regression parameters of a segmented linear model, characteristics of time series data need to be accounted for [17]. One of these characteristics is autocorrelation, which allows for the fact that values of near neighbouring datapoints may be more similar (or different) than distant datapoints [7, 18, 19]. If autocorrelation is unaccounted for [e.g., when using ordinary least squares (OLS), in the presence of (likely) positive autocorrelation] the regression parameter standard errors may be underestimated [17, 20, 21]. Several estimation methods are available to account for autocorrelation [e.g., restricted maximum likelihood (REML), Prais-Winsten (PW)] [20, 22, 23].

Two-stage meta-analysis may be used to combine effects across ITS studies. In the first stage, segmented linear models are fitted to each ITS study to obtain interruption effect estimates and their standard errors [24, 25]. These estimates may be reported in the primary publications, or the systematic reviewer may re-analyse the time series data to obtain the required estimates [26]. Then, in the second stage, the effect estimates are combined using a meta-analysis model; commonly either a fixed (common) effect or random-effects meta-analysis model [24]. Fixed-effect meta-analysis weights studies by the inverse of the variance of their estimated effect, and hence analysis requires only the effect estimates and their standard errors. However, the random-effects method weights additionally involve the between-study variance, a parameter which must be estimated and for which many estimators are available [24, 27–29]. Furthermore, there exist many confidence interval methods for the summary (combined) meta-analytic effect [30].

We previously undertook a numerical simulation study examining the performance of different meta-analysis methods to combine results from ITS studies with continuous outcomes, and how characteristics of the meta-analysis, ITS design, and method of analysis of the individual ITS studies modified the performance [31]. We examined ITS analysis and meta-analysis methods that are commonly used, or have been shown through numerical simulation to be preferable [20, 29, 30]. We found that all random-effects methods yielded confidence interval coverage for the summary effect close to the nominal level, irrespective of the ITS analysis method used. However, the between-study variance was overestimated in some scenarios [31]. In this companion study, we aimed to demonstrate empirically how the same methods compare when applied to real-world data, and answer the question: does statistical method choice importantly impact the meta-analysis results? Together, the simulation and empirical studies allow for a more complete understanding of which methods should be used in different scenarios. Specifically, our objectives were to: i) compare the meta-analysis estimates of the immediate level-change and slope-change, their standard errors, confidence intervals and *p*-values, and the estimates of between-study variance obtained from different meta-analysis and ITS analysis methods; and ii), create a repository of data from ITS studies.

## Methods

### Overview of the methods

**Fig. 2:**
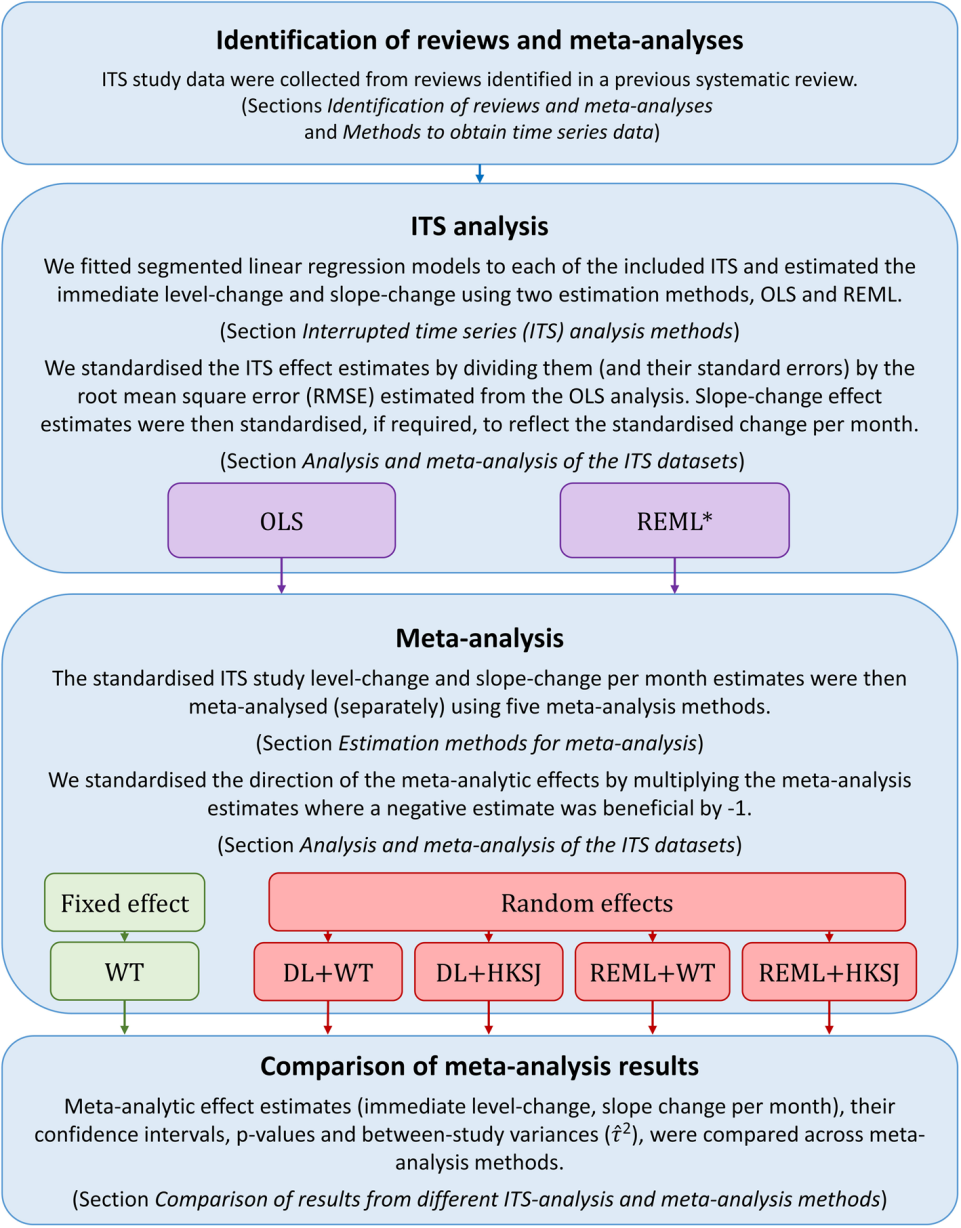
Depiction of the analysis methods used in this empirical study. *The estimation methods for ITS analysis are listed in order of preference, i.e. REML is used whenever it converges and the estimated autocorrelation is between -1 and 1, while PW followed by OLS are used in the case of non-convergence. *ITS* interrupted time series, *REML* restricted maximum likelihood, *PW* Prais-Winsten, *OLS* ordinary least squares, *DL* DerSimonian and Laird, *WT* Wald-type, *HKSJ* Hartung-Knapp/Sidik-Jonkman

An overview of the steps and corresponding Sections is depicted in Fig. 2. In brief, we sourced ITS data from published meta-analyses (sections *Identification of reviews and meta-analyses* and *Methods to obtain time series data*) and re-analysed them using two ITS analysis estimation methods (section *Interrupted time series (ITS) analysis methods*). The level-change and slope-change effect estimates (and their associated standard errors) were meta-analysed using a fixed-effect and four random-effects meta-analysis methods (section *Meta-analysis methods*). We compared the meta-analysis effect estimates, their standard errors, confidence intervals and *p*-values, and estimates of the between-study variance, across the meta-analysis methods (sections *Analysis and meta-analysis of the ITS datasets* and *Comparison of results from different ITS-analysis and meta-analysis methods*).

### Identification of reviews and meta-analyses

We sourced data for the present study from our previous methodological review that examined the statistical approaches used in reviews that include meta-analysis of ITS studies [26]. In brief, we searched eight electronic databases and included reviews containing at least one meta-analysis that included at least two ITS studies (using the review authors’ definition of an ITS). From each review, meta-analysis methods were examined for a single comparison-outcome (see the methodological review protocol for selection details [32]). In addition, reviews were eligible for the present study if:

1. The review’s meta-analysis included at least two ITS studies that had at least three datapoints before and after an interruption and a clearly defined interruption timepoint; and
2. The raw time series data were available. Data was classified as unavailable, if for example, the review authors had directly extracted effect estimates from the primary studies, or if it was not clear if the review authors had directly extracted effect estimates from the primary studies or re-analysed the raw time series data.

### Methods to obtain time series data

We sought the raw time series data using the following hierarchy of approaches:

1. Sourced the time series data from the review (e.g., where the data were available in supplementary files).
2. Contacted (via email) the corresponding author of the review, and requested the time points (and time unit, e.g., week, month), aggregate summary statistic (e.g., mean, rate, proportion), and time point(s) at which the interruption(s) occurred for each ITS.
3. Digitally extracted time series data from published figures in the review using WebPlotDigitizer [33]. This data extraction tool has been shown to yield data that can be used to obtain accurate estimates of the effect estimates and standard errors from published ITS graphs [34].

We only sought time series data from authors of the reviews, and not authors of the primary studies, for reasons of feasibility.

### Interrupted time series (ITS) analysis methods

#### Statistical model for an ITS analysis

We fitted the following segmented linear regression model to each of the included ITS studies [5]:

1 $${Y}_{t}={\beta }_{0}+{\beta }_{1}t+{\beta }_{2}{D}_{t}+{\beta }_{3}\left(t-{T}_{I}\right){D}_{t}+{\varepsilon }_{t}.$$

The continuous outcome at time $t (t=1, \dots , T)$ is represented by ${Y}_{t}$. The series are divided into two segments, before and after the interruption. The time of the interruption (*I*) occurs at time ${T}_{I}$. The segments are identified by ${D}_{t}$ (${D}_{t}={1}_{\left(t\ge {T}_{I}\right)}$ in the post-interruption period) (Additional file 1: Figure S1)$.$ ${\beta }_{0}$ represents the intercept in the pre-interruption period, ${\beta }_{1}$ the pre-interruption slope, and ${\beta }_{2}$ and ${\beta }_{3}$ represent the interruption effects—respectively, immediate level-change and slope-change. The error term accommodates lag-1 (AR(1)) autocorrelation ($\rho$) via ${\varepsilon }_{t}= \rho {\varepsilon }_{t-1}+{w}_{t}$, (${w}_{t}\sim N\left(\mathrm{0,1}\right)$); where $\rho {\varepsilon }_{t-1}$ allows for correlation between the current and the previous time point. Longer lags (i.e., higher order autocorrelation) can be modelled; however, we did not consider these here since we did not investigate longer lags in our companion numerical simulation study [31].

#### Estimation methods for ITS analysis

We used three statistical estimation methods for the analysis of the included ITS studies. These methods were selected because they are commonly used in practice [35], or have been shown to have improved statistical performance (via numerical simulation) [20, 22]. Briefly, the methods were:

- Ordinary least squares (OLS) [17], which assumes that the model errors are uncorrelated between observations. In the presence of positive autocorrelation, which has been shown to frequently occur in time series data [36], this assumption is violated, leading to potential underestimation of the variances of the regression parameters [15, 37];
- Prais-Winsten (PW), which is a generalised least-squares extension of OLS. PW estimation involves fitting the model using OLS and estimating lag-1 autocorrelation from the residuals, then, transforming the data using the estimated autocorrelation and re-estimating the regression parameters [23]. The aim is to remove the autocorrelation from the errors, which may require multiple iterations for the estimated autocorrelation to converge [23]. Accounting for autocorrelation in this way has been shown to improve estimation of the regression parameter standard errors compared with OLS estimation in the presence of autocorrelation; however, the standard errors are still underestimated using PW, particularly when there are few datapoints [20].
- Restricted Maximum Likelihood (REML), which is a form of maximum likelihood (ML) estimation, attempts to avoid the underestimation of the variance (and covariance) parameter estimates that can arise with ML estimation. REML involves separate estimation of the (co)variance parameters to account for the loss in degrees of freedom due to estimation of the regression parameters [22]. In the context of ITS studies, while both ML and REML directly estimate and adjust standard errors for autocorrelation, ML has been shown to yield less biased standard errors of the regression parameters compared with REML when autocorrelation was small, but positively biased standard errors when autocorrelation was large [20, 22].

### Meta-analysis methods

We used meta-analysis to combine the interruption effect estimates calculated using the methods in section *Interrupted time series (ITS) analysis methods* for each ITS study. We examined five meta-analysis methods, selected because they are frequently used in practice, or are known to have more favourable statistical properties.

#### Statistical models for meta-analysis

We examined a fixed-effect (common effect) and four random-effects models. The fixed-effect model is specified by:

2 $${\widehat{\beta }}_{mk}={\beta }_{m}+{\varepsilon }_{mk},$$

where it is assumed that each of the $K$ included ITS studies provide an estimate (${\widehat{\beta }}_{mk}$) of a single true interruption effect common to all studies, ${\beta }_{m}$ (where $m$ indicates the regression parameter of interest from Eq. 1, such as ${\beta }_{2}$ for immediate level-change), and any within-study error in the estimation is due to sampling variability alone, ${\varepsilon }_{mk}\sim N(0,{\sigma }_{mk}^{2}$).

The random-effects meta-analysis model is specified by:

3 $${\widehat{\beta }}_{mk}={\beta }_{m}^{*}+{\delta }_{mk}+{\varepsilon }_{mk}^{*},$$

where it is assumed that each of the $K$ ITS studies provide an estimate (${\widehat{\beta }}_{mk}$) of a true interruption effect specific to the ${k}^{th}$ study (i.e., ${\beta }_{m}^{*}+{\delta }_{mk}$), where ${\beta }_{m}^{*}$ represents the mean of the distribution of true interruption effects (for the ${m}^{th}$ regression parameter) and ${\delta }_{mk}$ represents a random effect in the ${k}^{th}$ ITS study, which are assumed to be normally distributed about the mean with a between-study variance ${\tau }_{m}^{2}$. The within-study error in estimating the ${k}^{th}$ ITS study’s interruption effect from a sample of participants is represented by ${\varepsilon }_{mk}^{*}\sim N(0,{\sigma }_{mk}^{2}$).

#### Estimation methods for meta-analysis

The meta-analytic effect of the ${m}^{th}$ regression parameter is calculated as a weighted average of the $K$ ITS study effect estimates, ${\widehat{\beta }}_{m}=\frac{\sum {W}_{mk}.{\widehat{\beta }}_{mk}}{\sum {W}_{mk}}$ (with a variance of $\frac{1}{\sum {W}_{mk}}$). The weight given to the ${k}^{th}$ ITS study is the reciprocal of the within-study variance, ${W}_{mkFE}=\frac{1}{{\sigma }_{mk}^{2}}$ when using a fixed-effect model, or ${W}_{mkRE}=\frac{1}{{\sigma }_{mk}^{2}+{\widehat{\tau }}_{m}^{2}}$ when using a random-effects model. Different between-study variance (${\widehat{\tau }}_{m}^{2})$ estimators are available [29], as well as methods to calculate the confidence interval for the meta-analytic effect [30]. We used two between-study variance estimators and two confidence interval methods.

We examined the following between-study variance estimators:

- DerSimonian and Laird (DL) [38], which is a moment-based between-study variance estimator derived from Cochran’s Q-statistic, was selected for evaluation in this study because it is commonly used in practice [26, 29]. However, DL is well known to yield biased estimates of the between-study variance in particular scenarios (i.e., small underlying between-study variance and few studies; or, many studies and large underlying heterogeneity) [31, 39, 40];
- Restricted Maximum Likelihood (REML), which is an iterative between-study variance estimator that attempts to correct for the negative bias associated with the ML estimator [29]. REML has been recommended as an alternative estimator because of its slightly improved performance compared with DL, and for this reason was selected for evaluation in this study [29, 40, 41].

We examined two confidence interval methods for the meta-analytic effect, which can be used with both the DL and REML between-study variance estimators:

- The Wald-type normal distribution (WT) confidence interval method [42], which uses the standard normal distribution to calculate the confidence limits. This method maintains the assumption of normality of ${\widehat{{\beta }^{*}}}_{m}$ despite the within-study and between-study variances not being known and instead estimated [28, 30]. The WT method relies on large-sample approximations, which are not generally met in the context of meta-analysis due to few included studies [43, 44]. This can lead to lower than nominal levels of 95% confidence interval coverage, particularly when there are few included studies or the between-study variance is large [30].
- The Hartung-Knapp [45]/Sidik-Jonkman [46] (HKSJ) confidence interval method, which attempts to overcome the assumption that the within-study variance is known and the between-study variance is accurately estimated, in scenarios where these conditions are unlikely to be met (e.g., meta-analyses with few studies of small sample sizes). The method involves making a small sample adjustment to the meta-analysis standard error and uses the t-distribution (with K-1 degrees of freedom) in the calculation of the confidence limits. This adjustment yields wider confidence intervals than the WT method, except when there are few studies and the estimated between-study variance is zero [29].

### Analysis and meta-analysis of the ITS datasets

Prior to fitting the models, we excluded ITS from the meta-analyses where the study i) did not meet our minimum required number of datapoints, or ii) had a large proportion of time series datapoints that were zero (i.e., greater than 40%), such that it was not reasonable to assume that the error term would be normally distributed. In addition, we removed any control series that were included in the original meta-analysis, because our interest was in the interrupted series only. Furthermore, we excluded segments of studies that had multiple interruptions. Specifically, we only included the first interruption (and the adjacent segments). Additional file 1: Table S1 includes all modifications, with justifications. Modifications were discussed and agreed upon at team meetings (including authors EK, SLT, ABF, AK and JEM).

We fitted a segmented linear regression model (section *Statistical model for an ITS analysis*, Eq. 1) to each ITS study and estimated the regression parameters (immediate level-change (${\beta }_{2}$) and slope-change (${\beta }_{3}$)) using both OLS and REML (section *Estimation methods for ITS analysis*) (Fig. 2). If REML failed to converge or to yield an estimate of autocorrelation between -1 and 1, we used PW, and where PW failed, we used OLS. Given the outcomes varied across the meta-analyses, we standardised the ITS study effect estimates (immediate level-change, slope-change) prior to meta-analysis, so that the resulting meta-analysis effect estimates were standardised and comparable across meta-analyses. The ITS effect estimates obtained via REML, PW and OLS were standardised by dividing them (and their standard errors) by the root mean square error estimated from the OLS analysis. Slope-change effect estimates were then standardised, if required, to reflect the standardised slope-change *per month* by multiplying or dividing by an appropriate factor (e.g., slope-change calculated from a series with yearly timepoints was divided by 12 to reflect the slope-change per month).

The standardised ITS study level-change and slope-change estimates were then meta-analysed (separately) using five meta-analysis methods (section *Estimation methods for meta-analysis*; Fig. 2). We standardised the direction of these meta-analysis effects so that for all a positive estimate reflected a beneficial impact of the interruption. This was achieved by multiplying the meta-analysis estimates where a negative estimate was beneficial (e.g., a decrease in fatality rates) by -1, to reverse the direction of interpretation.

We undertook sensitivity analyses to investigate whether the results were robust to our choice of threshold for excluding ITS based on the proportion of datapoints that were zero. For the sensitivity analysis, we excluded ITS from the meta-analyses where the study had greater than 30% but less 40% of time series datapoints that were zero. We then repeated the above analyses and informally compared the results.

All analyses were performed using Stata version 16.1 [47] and results were visualised using R version 4.1.0 (*dplyr* [48]*, foreign* [49]*, ggplot2* [50]). Code and the repository of data are available in the Monash University online repository, Bridges [51].

### Comparison of results from different ITS-analysis and meta-analysis methods

We compared meta-analysis effect estimates (i.e., immediate level-change and slope-change), and their standard errors between each of the combinations of ITS analysis methods and meta-analysis methods. For each pairwise comparison between the combinations, we calculated (and tabulated) the average of the differences between the estimates (i.e., the mean difference = the sum of the differences between the estimates yielded by the two methods being compared, divided by the number of meta-analyses, 17) and the limits of agreement (calculated as the mean difference ± 1.96 × standard deviation of the differences) [52]. The limits of agreement provide a range within which most of the differences between estimates will lie [52]. For the standard errors, we first log-transformed these to remove the relationship between the variability of the differences and the magnitude of the standard errors [52]. We used Bland–Altman scatter plots to visualise the agreement, whereby, for each pairwise comparison between combinations, we plotted the difference between the estimates vs their average [52].

We compared confidence interval widths between each of the combinations of ITS analysis and meta-analysis methods. For each pairwise comparison, we plotted the ratio of the confidence intervals, scaled such that the reference confidence interval width spanned -0.5 to 0.5 (following the approach of Turner et al. [36]).

We compared the estimates of between-study variance (${\widehat{\tau }}^{2}$) between each combination of ITS analysis methods and between-study variance estimators. For each meta-analysis and pairwise comparison, we calculated (and tabulated) the median and interquartile range (IQR) of the differences between the estimates of the between-study variance.

We compared the *p*-values of the meta-analytic level-change and slope-change estimates between each of the combinations of ITS analysis and meta-analysis methods. We categorised the *p*-values using the conventionally used (though not recommended) statistical significance threshold of 0.05. The percentage of meta-analyses where there was agreement in the categories of statistical significance was calculated. Namely, the percentage of meta-analyses where the *p*-value for the effect estimate from both methods was < 0.05 or $\ge$ 0.05. Agreement between the statistical methods in the conclusion about the statistical significance was further quantified using the kappa statistic, where we used the following adjectives to describe agreement: *moderate* agreement as a kappa value of 0.41–0.6, *substantial* agreement as a value of 0.61–0.8, and *almost perfect* agreement as a value of 0.81–1.0 [53].

## Results

**Fig. 3:**
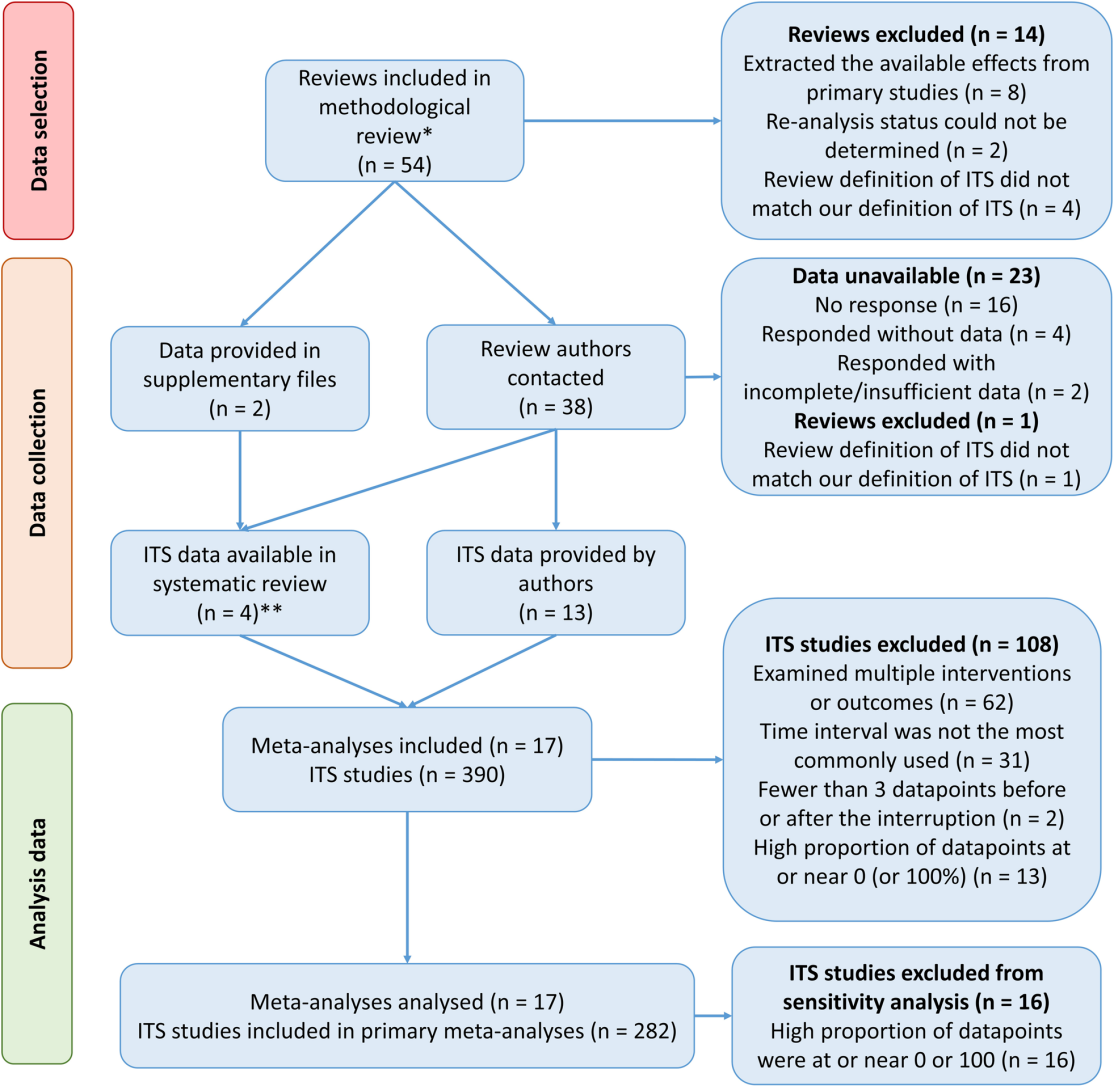
Flow diagram of included reviews, their meta-analysis and interrupted time series (ITS) studies. *54 reviews were identified in a methodological systematic review (see Korevaar et al. [26] for the search strategy used). **Two authors that were contacted did not provide data, as such, we digitally extracted the raw time series data from the figures provided in the review manuscripts

Of the 54 reviews included in the source methodological review [26], 40 met the additional eligibility criteria for the present study (Fig. 3). We extracted data from the supplementary material of two reviews, and emailed the remaining 38 review authors. Of these, 35 emails were successfully delivered, from which 13 authors provided data. For a further two reviews, it was possible to digitally extract data from the ITS graphs included in the reviews. This resulted in the inclusion of 17 meta-analyses with 390 ITS. We further excluded 108 ITS from these meta-analyses for a variety of reasons (Fig. 3), leaving 282 ITS (from 17 meta-analyses) for our primary analyses.

### Characteristics of the included meta-analyses and ITS studies

The reviews were published between 2005 and 2019. Most reviews investigated the effects of public health interruptions (88%, 15/17) [e.g., examining the impact of insecticide space spraying strategies on the incidence of malaria], while two examined the effects of crime interventions (12%, 2/17) (Table 1). The interruptions were predominantly targeted at the population level (59%, 10/17) [e.g., state-wide legislation] or organisational level (30%, 5/17) [e.g., hospital-wide policy]. The 17 included meta-analyses had a median of 11 included ITS studies (IQR: 5.0–15.0, range: 3–62). The median series length of the ITS studies was 52 (IQR: 27–61, range: 7–195, *n* = 282), while the average series length at the meta-analysis level had a median of 40 (IQR: 22–59, range: 9.7–165.3). The time interval used for aggregation of the datapoints was most commonly months (11/17, 65%) followed by years (4/17, 24%). The outcome types were predominantly rates (6/17, 35%) and counts (5/17, 29%). The autocorrelation of the ITS studies estimated by REML ITS analysis had a median of 0.22 (IQR: 0.00, 0.48, *n* = 282), while the average estimate of autocorrelation at the meta-analysis level had a median of 0.17 (IQR: 0.13, 0.42).

**Table 1:** Characteristics of included meta-analyses and ITS studies

		**Meta-analyses** **(*N* = 17)** **n (%) or median (IQR)**
**Discipline/Topic**^**a**^	Public health	15 (88)
	Crime	2 (12)
**Interruption target**	Population	10 (59)
	Organisation	5 (30)
	Individual^b^	1 (6)
	Combination	1 (1)
**Interruption types**^**a**^	Policy change	12 (71)
	Practice change	5 (39)
	Communication (campaign)	3 (18)
	Educational method	3 (18)
	Exposure	2 (12)
**Outcome types**^**a**^	Rate	6 (35)
	Count	5 (29)
	Proportion	2 (12)
	Combination ^c^	2 (12)
	Continuous	1 (6)
	Probability	1 (6)
**Number of ITS studies**	Per meta-analysis	11 (5, 15)
**Number of time series datapoints**	ITS level (*N* = 282)	52 (27, 61)
	Meta-analysis level^d^	40 (22, 53)
**Autocorrelation**^**e**^	ITS level (*N* = 282)	0.22 (0.00, 0.48)
	Meta-analysis level^d^	0.17 (0.13, 0.42)
**Time interval for time series datapoints**	Month	11 (65)
	Year	4 (24)
	4-weeks	1 (6)
	Day	1 (6)

*ITS* interrupted time series, *IQR* interquartile range, *PW* Prais-Winsten, *REML* restricted maximum likelihood

^a^Multiple response options possible therefore percentages sum to greater than 100%

^b^Interruptions classified as ‘individual-level interruption’ were the intervention directed at an individual (e.g., delivery of a vaccine), however, the measurements were still aggregated over units of time (e.g., number of vaccinations each year)

^c^Combination indicates where reviewers combined multiple data types (e.g., combining studies using proportion and rate outcomes)

^d^The average number of datapoints and autocorrelation were calculated across the series included in each meta-analysis. These averages were then summarised across the meta-analyses using the median and IQR

^e^Autocorrelation estimated using REML (or PW where REML failed to converge)

### Convergence of ITS analyses and meta-analyses using REML

Of the 282 ITS that were analysed using REML, 255 (90%) converged. For those that did not converge, PW was used, of which 4/27 (19%) failed to converge. OLS was used for the four that did not converge. All meta-analyses using REML converged.

### Comparison of results from the different meta-analysis and ITS analysis method combinations

#### Estimates of level- and slope-change meta-analytic effect estimates

**Fig. 4:**
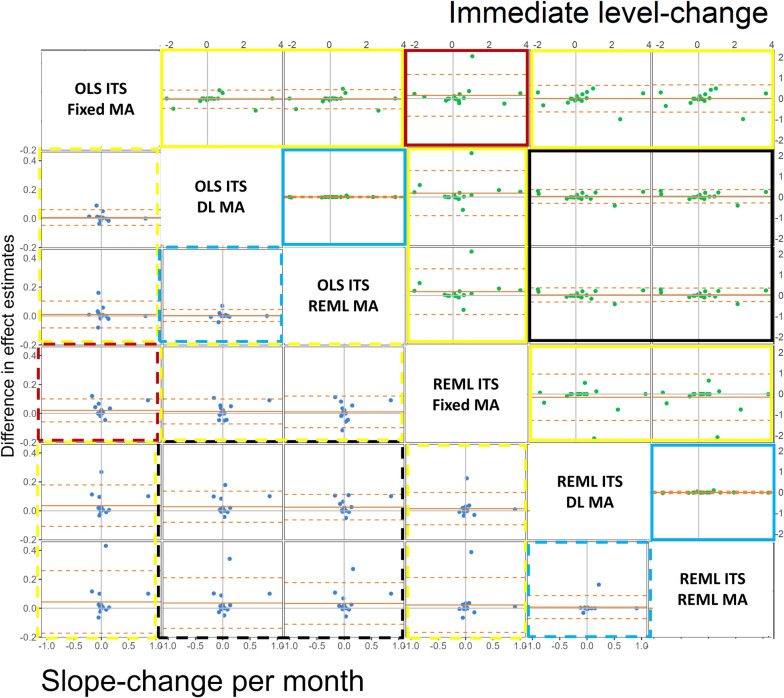
Bland Altman plot of difference in standardised meta-analytic effect estimates (y-axis) vs average of the effect estimates (x-axis), for each pairwise comparisons of ITS analysis and meta-analysis method combination (top row of the label indicates the ITS analysis methods, bottom row indicates the meta-analysis method, e.g., OLS ITS DL MA is OLS ITS analysis with DerSimonian and Laird between-study variance meta-analysis). The top triangle (green points) presents the immediate level-change (difference calculated as column method – row method), and the bottom triangle (blue points) presents the slope-change per month (difference calculated as row method – column method). Horizontal orange lines depict the average, dashed orange lines depict the 95% limits of agreement (calculated as the mean ± 1.96*standard deviation of the differences). Vertical grey line indicates an average of zero, while the horizontal grey line indicates a mean difference of zero. The coloured boxes indicate cells that compare ITS analysis methods when fixed-effect meta-analysis was fitted (red boxes), meta-analysis models (i.e., fixed- vs random-effects models)[yellow boxes], between-study variance estimators (i.e., using DL or REML)[blue boxes], and combinations of between-study variance estimators and ITS analysis methods (black boxes). The solid coloured boxes indicate comparisons of level-change and dashed boxes indicate slope-change per month. *DL* DerSimonian and Laird, *HKSJ* Hartung-Knapp/Sidik-Jonkman, *ITS* interrupted time series, *MA* meta-analysis, *OLS* ordinary least squares, *REML* restricted maximum likelihood, *WT* Wald-type

When fixed-effect meta-analysis was fitted, on average, REML ITS analysis yielded slightly larger estimated immediate level-changes compared with OLS (depicted by the horizontal solid orange line, representing the average of the differences, being greater than zero in Fig. 4, solid red box; Table 2), but with wide limits of agreement (depicted by the horizontal dashed orange lines being wide), largely due to the influence of one outlying estimated level-change using REML. The different between-study variance estimators (i.e., using DL or REML) had no impact on the immediate level-change within ITS analysis method (i.e., OLS ITS analysis with the DL between-study variance estimator vs OLS ITS with the REML estimator; REML ITS analysis with the DL between-study variance estimator vs REML ITS with the REML estimator), as depicted by the horizontal solid orange line sitting on zero, and the limits of agreement being close to zero in Fig. 4 (solid blue boxes). Furthermore, the estimated meta-analytic immediate level-changes were, on average, similar across the combinations of between-study variance estimators and ITS analysis methods (Fig. 4 solid black boxes); however, the limits of agreement (which were approximately $\pm 0.33$) showed that methods could yield small to moderate differences in estimates of level-change for a given meta-analysis. The patterns were similar for the effect estimates of the meta-analytic slope-change per month (see Fig. 4, dashed boxes).

**Table 2:** The mean difference of effect estimates and 95% limits of agreement for the meta-analytic immediate level-change (top triangle, difference calculated as column method – row method) and slope-change per month (bottom triangle, difference calculated as row method – column method) (*n* = 17)

	**Level-change**					
**Slope-change per month**	**OLS ITS** **Fixed MA**	-0.04 (-0.48,0.40)	-0.034 (-0.50,0.43)	0.15 (-0.85,1.15)	-0.01 (-0.65,0.63)	0.00 (-0.66,0.66)
	0.01 (-0.05,0.06)	**OLS ITS** **DL MA**	0.00 (-0.04,0.04)	0.19 (-0.90,1.27)	0.03 (-0.30,0.36)	0.04 (-0.29,0.36)
	0.01 (-0.08,0.10)	0.00 (-0.04,0.04)	**OLS ITS** **REML MA**	0.19 (-0.92,1.29)	0.03 (-0.31,0.37)	0.03 (-0.29,0.36)
	0.02 (-0.06,0.10)	0.01 (-0.07,0.10)	0.01 (-0.10,0.12)	**REML ITS** **Fixed MA**	-0.16 (-1.28,0.96)	-0.15 (-1.27,0.96)
	0.04 (-0.11,0.18)	0.03 (-0.08,0.14)	0.03 (-0.06,0.11)	0.01 (-0.10,0.13)	**REML ITS** **DL MA**	0.01 (-0.05,0.07)
	0.04 (-0.17,0.26)	0.04 (-0.14,0.21)	0.03 (-0.11,0.18)	0.02 (-0.17,0.21)	0.01 (-0.07,0.09)	**REML ITS** **REML MA**

The top row of the label indicates the ITS analysis methods, bottom row indicates the meta-analysis method, e.g., OLS Fixed is OLS ITS analysis and fixed-effect meta-analysis. For example, the mean meta-analytic level-change yielded by REML ITS analysis with fixed-effect meta-analysis was 0.15 higher than that yielded by OLS ITS analysis with fixed-effect meta-analysis (column 4, row 1). The mean meta-analytic slope-change per month yielded by REML ITS analysis with fixed-effect meta-analysis was 0.02 higher than that yielded by OLS ITS analysis with fixed-effect meta-analysis (column 1, row 4)

*DL* DerSimonian and Laird, *ITS* interrupted time series, *MA *meta-analysis, *OLS* ordinary least squares, *REML* restricted maximum likelihood

#### Standard errors of the level- and slope-change meta-analytic effects

**Fig. 5:**
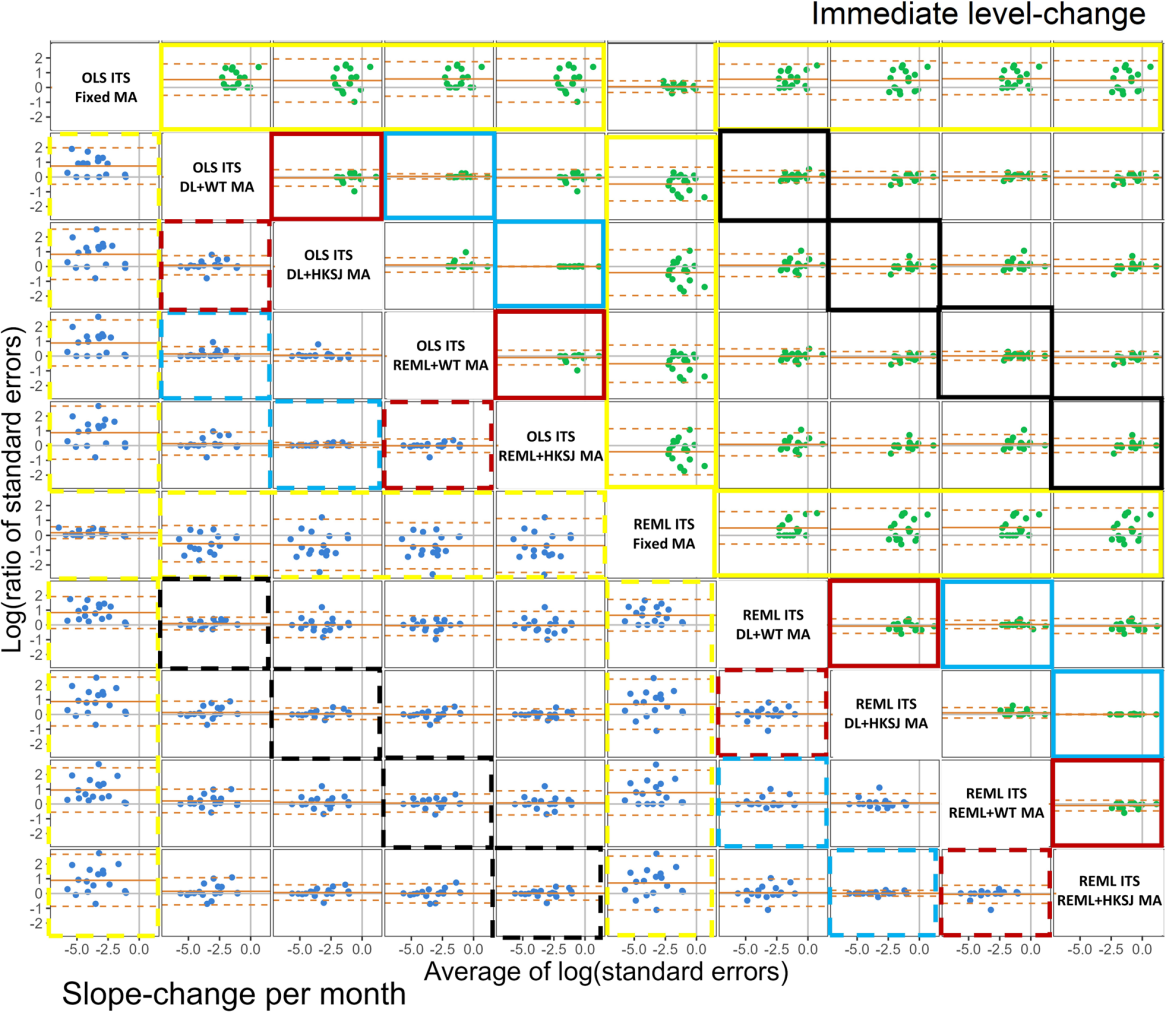
Bland Altman plot of log ratio of standard errors of the standardised meta-analytic effect estimates (y-axis) vs average of the standard errors (x-axis), for each pairwise comparisons of ITS analysis and meta-analysis method combination (top row of the label indicates the ITS analysis methods, bottom row indicates the meta-analysis method, e.g., OLS ITS DL MA is OLS ITS analysis with DerSimonian and Laird between-study variance meta-analysis). The top triangle (green points) presents the immediate level-change [log ratio calculated as log(column method / row method)], and the bottom triangle (blue points) presents the slope-change per month [log ratio calculated as log(row method / column method)]. Horizontal orange lines depict the average, dashed orange lines depict the 95% limits of agreement (calculated as the mean ± 1.96*standard deviation of the log(ratio)). Vertical grey line indicates an average of zero, while the horizontal grey line indicates a log(ratio) of zero. The coloured boxes indicate cells that compare meta-analysis models (i.e., fixed- vs random-effects models)[yellow boxes], ITS analysis methods when random-effects meta-analysis was used (black boxes), between-study variance estimators (blue boxes) and confidence interval methods (red boxes). The solid coloured boxes indicate comparisons of level-change and dashed boxes indicate slope-change per month. *DL* DerSimonian and Laird, *HKSJ* Hartung-Knapp/Sidik-Jonkman, *ITS* interrupted time series, *OLS* ordinary least squares, *REML* restricted maximum likelihood, *WT* Wald-type

The standard errors of the meta-analytic level-change were most influenced by the meta-analysis model, with the standard errors being substantially larger when a random-effects model was fitted (as depicted by the horizontal solid orange line being greater or less than zero, depending on the order of the comparisons, in Fig. 5, yellow boxes, and Table 3). When random-effects meta-analysis methods were fitted, on average, there were no important differences in the standard errors of the meta-analytic level-change (depicted by the horizontal solid orange line sitting on zero in Fig. 5), across ITS analysis methods (black boxes), between-study variance estimators (blue boxes) or where there was a small sample adjustment made to the meta-analysis standard error (as occurs with the HKSJ method)[red boxes]. However, the limits of agreement were wide across ITS analysis methods (black boxes) and where there was a small sample adjustment (red boxes); for example, the limits of agreement for the comparison of REML ITS vs OLS ITS analysis (both with the REML between-study variance estimator and HKSJ confidence interval method) suggest that the meta-analysis estimate of standard error is likely to be between 37% smaller to 63% larger when using REML ITS compared with OLS ITS analysis (Table 3). The patterns were similar for the standard errors of the meta-analytic slope-change per month (dashed boxes).

**Table 3:** The mean ratio of standard errors and 95% limits of agreement for the meta-analytic immediate level-change (top triangle, ratio calculated as column method / row method) and slope-change per month (bottom triangle, ratio calculated as row method / column method) (*n* = 17)

	**Level-change**									
**Slope-change per month**	**OLS ITS** **Fixed MA**	1.70 (0.59,4.94)	1.60 (0.37,6.90)	1.78 (0.55,5.73)	1.61 (0.37,6.99)	1.06 (0.71,1.57)	1.75 (0.63,4.85)	1.61 (0.43,6.02)	1.80 (0.61,5.32)	1.62 (0.43,6.12)
	2.10 (0.61,7.22)	**OLS ITS** **DL + WT MA**	0.94 (0.54,1.64)	1.04 (0.88,1.24)	0.94 (0.53,1.67)	0.62 (0.20,1.95)	1.03 (0.68,1.55)	0.95 (0.61,1.46)	1.06 (0.79,1.41)	0.95 (0.61,1.48)
	2.29 (0.42,12.57)	1.09 (0.57,2.09)	**OLS ITS** **DL + HKSJ MA**	1.11 (0.68,1.82)	1.00 (0.98,1.02)	0.66 (0.14,3.11)	1.09 (0.51,2.35)	1.01 (0.62,1.63)	1.13 (0.60,2.11)	1.01 (0.63,1.63)
	2.42 (0.51,11.52)	1.15 (0.70,1.90)	1.06 (0.71,1.58)	**OLS ITS** **REML + WT MA**	0.90 (0.55,1.48)	0.59 (0.17,2.11)	0.98 (0.59,1.63)	0.91 (0.61,1.36)	1.01 (0.75,1.37)	0.91 (0.61,1.36)
	2.40 (0.40,14.49)	1.14 (0.52,2.49)	1.05 (0.89,1.24)	0.99 (0.62,1.56)	**OLS ITS** **REML + HKSJ MA**	0.66 (0.14,3.13)	1.09 (0.50,2.37)	1.00 (0.62,1.63)	1.12 (0.60,2.11)	1.01 (0.62,1.63)
	1.20 (0.80,1.79)	0.57 (0.17,1.95)	0.52 (0.09,2.94)	0.49 (0.10,2.35)	0.50 (0.08,3.08)	**REML ITS** **Fixed MA**	1.65 (0.56,4.90)	1.52 (0.38,6.15)	1.70 (0.53,5.45)	1.53 (0.37,6.28)
	2.31 (0.78,6.82)	1.10 (0.72,1.68)	1.01 (0.42,2.40)	0.95 (0.49,1.87)	0.96 (0.37,2.50)	1.93 (0.66,5.62)	**REML ITS** **DL + WT MA**	0.92 (0.56,1.51)	1.03 (0.78,1.37)	0.93 (0.56,1.54)
	2.40 (0.46,12.52)	1.14 (0.53,2.46)	1.05 (0.71,1.55)	0.99 (0.57,1.70)	1.00 (0.69,1.46)	2.00 (0.37,10.96)	1.04 (0.46,2.34)	**REML ITS** **DL + HKSJ MA**	1.12 (0.78,1.59)	1.01 (0.98,1.03)
	2.58 (0.58,11.57)	1.23 (0.55,2.73)	1.13 (0.50,2.53)	1.06 (0.58,1.96)	1.08 (0.48,2.44)	2.15 (0.47,9.88)	1.12 (0.60,2.08)	1.08 (0.57,2.02)	**REML ITS** **REML + WT MA**	0.90 (0.63,1.29)
	2.44 (0.42,14.23)	1.16 (0.46,2.92)	1.07 (0.63,1.80)	1.01 (0.53,1.93)	1.02 (0.64,1.63)	2.04 (0.33,12.60)	1.06 (0.42,2.67)	1.02 (0.84,1.24)	0.95 (0.51,1.75)	**REML ITS** **REML + HKSJ MA**

For example, on average the standard error of the meta-analytic level-change yielded by REML ITS analysis with fixed effect meta-analysis was 6% higher than the standard error yielded by OLS ITS analysis with fixed effect meta-analysis (column 6, row 1), while the standard error of the meta-analytic slope-change per month yielded by REML ITS analysis with fixed effect meta-analysis was, on average, 20% higher than the standard error yielded by OLS ITS analysis with fixed effect meta-analysis (column 1, row 6)

*DL* DerSimonian and Laird, *HKSJ* Hartung-Knapp/Sidik-Jonkman, *ITS* interrupted time series, *MA* meta-analysis, *OLS* ordinary least squares, *REML* restricted maximum likelihood, *WT* Wald-type

#### Confidence intervals of level- and slope-change meta-analytic effects

**Fig. 6:**
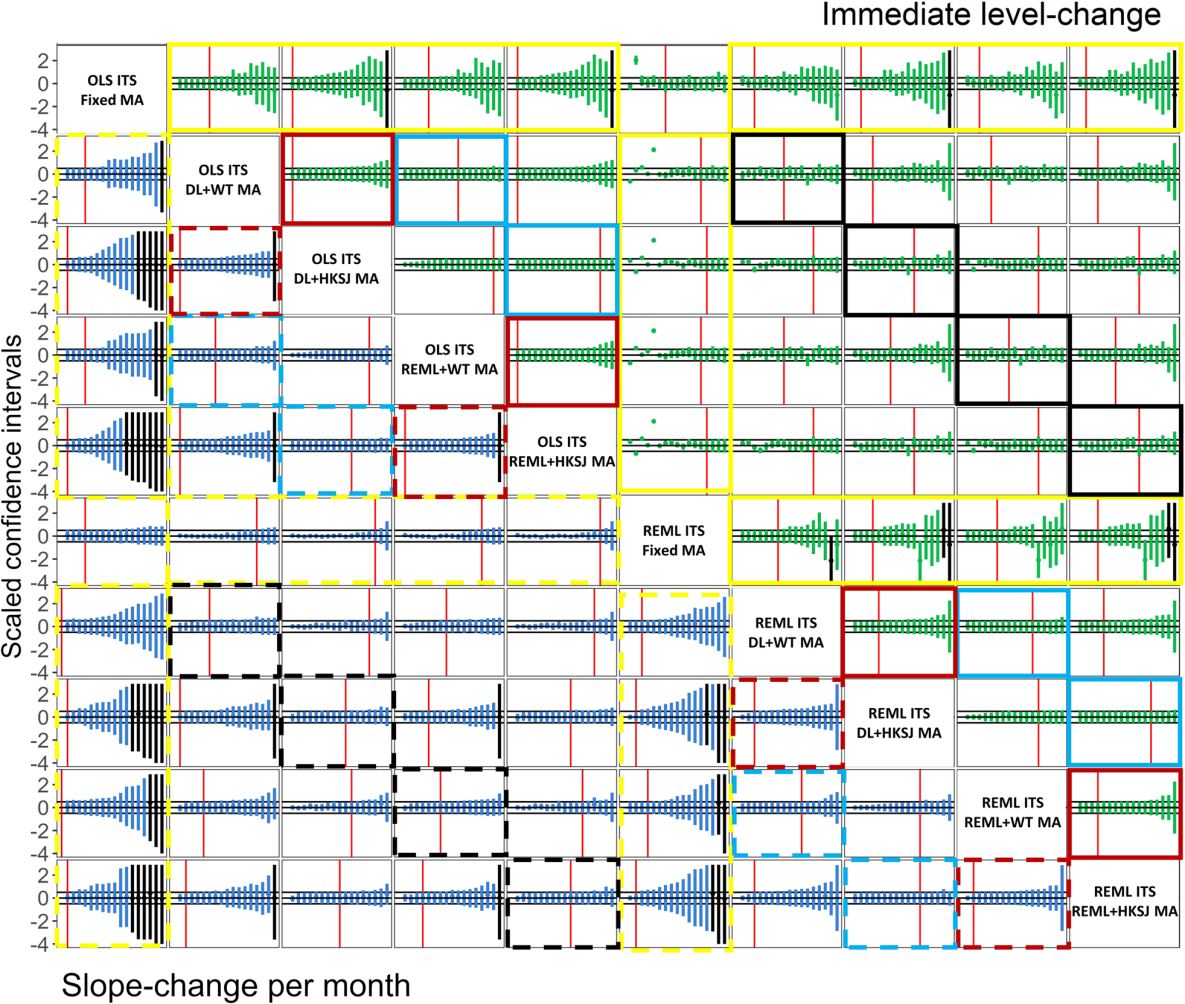
Pairwise comparison of confidence intervals yielded by combinations of ITS analysis (OLS or REML) and meta-analysis methods (fixed, DL + WT, DL + HKSJ, REML + WT or REML + HKSJ). Each plot contains the 17 meta-analyses’ absolute difference in meta-analytic effect estimates and scaled relative confidence intervals, ranked in order of scaled relative confidence interval width. The top triangle (green points) presents the immediate level-change, while the bottom triangle (blue points) presents the slope-change per month. The scaled relative confidence interval widths for the level-change were calculated as column method confidence interval width divided by row method confidence interval width (and row method / column method for slope-change per month), scaled such that the row method (column method in the case of slope-change per month) spans -0.5 to 0.5 (indicated by the horizontal grey lines, which form the ‘reference rectangle’). Confidence intervals entirely within the reference rectangle (i.e., between the horizontal grey lines) have smaller confidence intervals than the comparison (left of the vertical red line), while the confidence intervals extending beyond the reference rectangle have larger confidence intervals than the comparison (right of the vertical red line). The black confidence intervals indicate where one or both of the confidence limits were beyond the limits y-axis scale. The coloured boxes indicate cells that compare meta-analysis models (i.e., fixed- vs random-effects models)[yellow boxes], ITS analysis methods when random-effects meta-analysis was used (black boxes), between-study variance estimators (blue boxes) and confidence interval methods (red boxes). The solid coloured boxes indicate comparisons of level-change and dashed boxes indicate slope-change per month. See Additional file 1: Figure S3 for random-effect meta-analysis comparisons only. *DL* DerSimonian and Laird, *HKSJ* Hartung-Knapp/Sidik-Jonkman, *ITS* interrupted time series, *OLS* ordinary least squares, *REML* restricted maximum likelihood, *WT* Wald-type

The confidence interval widths of the random-effects meta-analytic level-change were similar irrespective of the ITS analysis method, or between-study variance estimator (as depicted by the confidence intervals being the width of the reference rectangle in Fig. 6 black and blue boxes, see Additional file 1: Figure S3 for random-effect meta-analysis comparisons only). However, the confidence interval widths were mostly similar or wider when the HKSJ method was used as compared with the WT confidence interval method (as depicted by confidence intervals being the width of the reference rectangle, or wider, in Fig. 6 red boxes). The confidence intervals of the random-effects meta-analytic slope-change per month were more variable than the level-change confidence interval widths; however, the patterns were the same (dashed boxes).

#### *p*-values

Pairwise comparisons of the meta-analytic level-change statistical significance between REML ITS analysis and OLS ITS analysis (keeping meta-analysis method constant) ranged from substantial to almost perfect agreement, irrespective of the meta-analysis methods used (Table 4 and Additional file 1: Table S2). Similarly, the level of statistical significance agreement between comparisons of between-study variance estimators, and comparisons of confidence interval methods, ranged from substantial to almost perfect agreement. However, the agreement was systematically (slightly) lower when REML ITS analysis was used compared to OLS. In addition, the statistical significance agreement was lower when different confidence interval methods were used; this reduction in agreement was more pronounced when REML ITS analysis was used compared with OLS. The patterns were similar for the statistical significance agreement for the meta-analytic slope-change per month, which ranged from moderate to almost perfect agreement between most pairwise comparisons, irrespective of the statistical methods used.

**Table 4:** The percentage agreement (kappa statistic) in statistical significance (categorised as p $\le$ 0.05 or *p* > 0.05) of level-change (upper triangle) and slope-change per month effect estimates (lower triangle, shaded grey) when ITS are analysed with OLS and REML, and meta-analysed by fixed, DL + WT and REML + HKSJ meta-analysis methods (*n* = 17)

	**Level-change**									
**Slope-change per month**	**OLS ITS** **Fixed MA**	70.6% (0.43)	64.7% (0.32)	70.6% (0.43)	64.7% (0.32)	82.4% (0.65)	82.4% (0.65)	70.6% (0.43)	76.5% (0.54)	64.7% (0.32)
	76.5% (0.55)	**OLS ITS** **DL + WT MA**	94.1% (0.82)	100.0% (1.00)	94.1% (0.82)	76.5% (0.51)	88.2% (0.72)	100.0% (1.00)	94.1% (0.85)	94.1% (0.82)
	64.7% (0.35)	88.2% (0.72)	**OLS ITS** **DL + HKSJ MA**	94.1% (0.82)	100.0% (1.00)	70.6% (0.39)	82.4% (0.56)	94.1% (0.82)	88.2% (0.68)	88.2% (0.60)
	64.7% (0.35)	88.2% (0.72)	88.2% (0.67)	**OLS ITS** **REML + WT MA**	94.1% (0.82)	76.5% (0.51)	88.2% (0.72)	100.0% (1.00)	94.1% (0.85)	94.1% (0.82)
	58.8% (0.26)	82.4% (0.56)	94.1% (0.82)	94.1% (0.82)	**OLS ITS** **REML + HKSJ MA**	70.6% (0.39)	82.4% (0.56)	94.1% (0.82)	88.2% (0.68)	88.2% (0.60)
	82.4% (0.66)	82.4% (0.63)	70.6% (0.35)	70.6% (0.35)	64.7% (0.20)	**REML ITS** **Fixed MA**	76.5% (0.52)	76.5% (0.51)	82.4% (0.64)	70.6% (0.39)
	64.7% (0.35)	88.2% (0.72)	88.2% (0.67)	88.2% (0.67)	82.4% (0.46)	82.4% (0.61)	**REML ITS** **DL + WT MA**	88.2% (0.72)	94.1% (0.87)	82.4% (0.56)
	58.8% (0.26)	82.4% (0.56)	94.1% (0.82)	94.1% (0.82)	100.0% (1.00)	64.7% (0.20)	82.4% (0.46)	**REML ITS** **DL + HKSJ MA**	94.1% (0.85)	94.1% (0.82)
	58.8% (0.26)	82.4% (0.56)	82.4% (0.46)	94.1% (0.82)	88.2% (0.60)	76.5% (0.47)	94.1% (0.82)	88.2% (0.60)	**REML ITS** **REML + WT MA**	88.2% (0.68)
	58.8% (0.26)	82.4% (0.56)	94.1% (0.82)	94.1% (0.82)	100.0% (1.00)	64.7% (0.20)	82.4% (0.46)	100.0% (1.00)	88.2% (0.60)	**REML ITS** **REML + HKSJ MA**

The top row of the label indicates the ITS analysis methods, bottom row indicates the meta-analysis method, e.g., OLS Fixed is OLS ITS analysis and fixed effect meta-analysis. We used the following adjectives to describe agreement: moderate agreement as a kappa value of 0.41–0.6, substantial agreement as a value of 0.61–0.8, and almost perfect agreement as a value of 0.81–1.0

*DL* DerSimonian and Laird, *HKSJ* Hartung-Knapp/Sidik-Jonkman, *ITS* interrupted time series, *MA* meta-analysis, *OLS* ordinary least squares, *REML* restricted maximum likelihood, *WT* Wald-type

#### Estimates of between-study variance

**Fig. 7:**
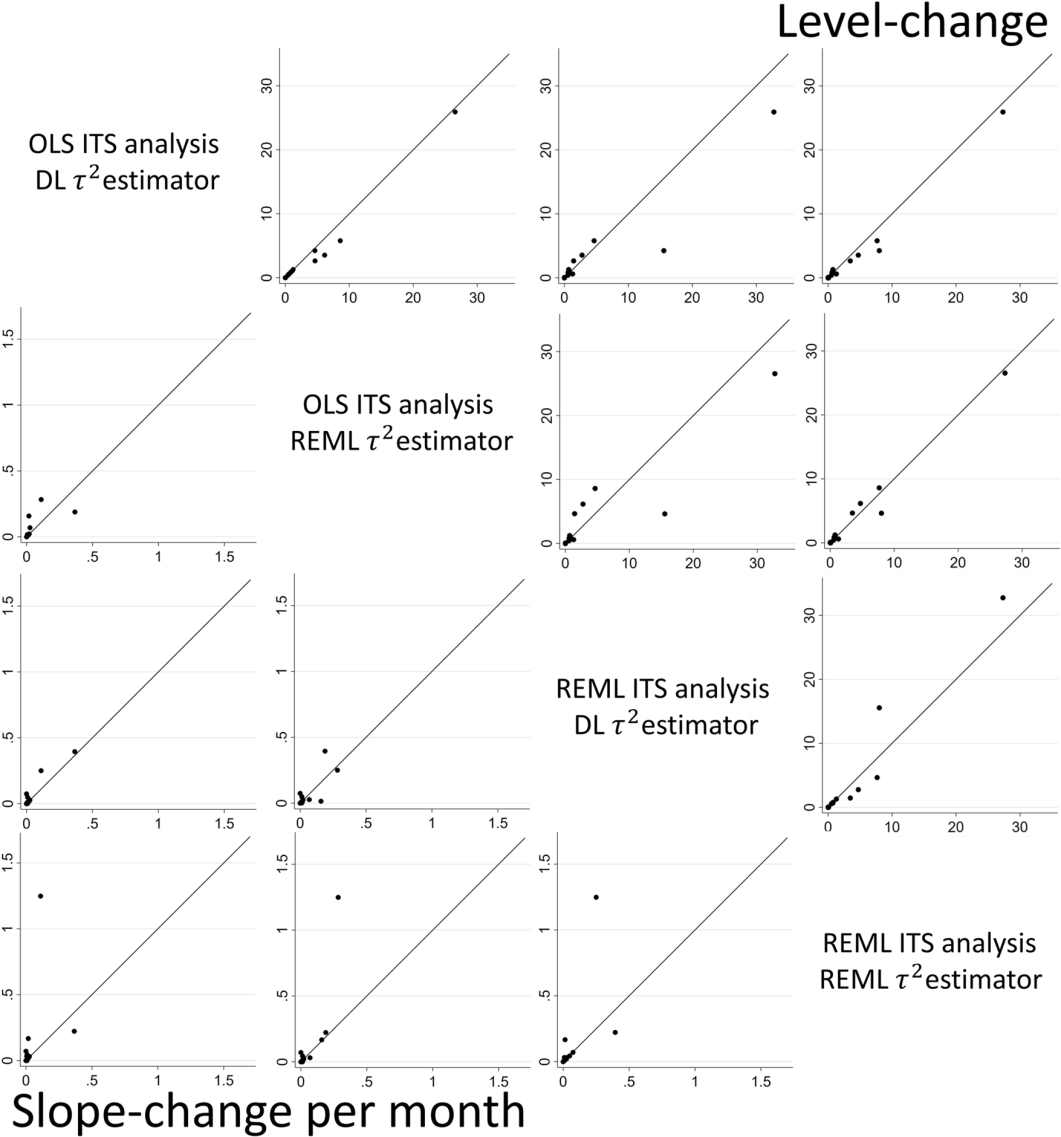
Pairwise comparisons of the between-study variance estimates (${\widehat{\tau }}^{2}$) yielded by combinations of ITS analysis methods and between-study variance estimators. The between-study variance estimate yielded by the row method (y-axis) versus the between-study variance estimate yielded by the column method (x-axis), for the level-change (top triangle) and slope-change per month (bottom triangle) meta-analyses. *DL* DerSimonian and Laird, *HKSJ* Hartung-Knapp/Sidik-Jonkman,* ITS* interrupted time series, *OLS* ordinary least squares, *REML* restricted maximum likelihood

We compared the between-study variance estimates yielded by different combinations of ITS analysis methods (OLS and REML) and the between-study variance estimators (DL and REML). The median and IQR of the pairwise differences in between-study variance estimates indicated no substantial differences (Fig. 7 and Table 5).

**Table 5:** The median and IQR for the differences in between-study variance estimates for the meta-analytic immediate level-change (top triangle, difference calculated as column method – row method) and slope-change per month (bottom triangle, difference calculated as row method – column method) (*n* = 17)

	**Level-change**			
**Slope-change per month**	**OLS ITS** **DL MA**	0.00 (0.00,0.38)	0.00 (-0.28,0.00)	0.08 (0.00,0.83)
	0.00 (0.00,0.00)	**OLS ITS** **REML MA**	0.00 (-0.29,0.00)	0.00 (-0.29,0.08)
	0.00 (0.00,0.01)	0.00 (-0.01,0.00)	**REML ITS** **DL MA**	0.00 (0.00,0.08)
	0.00 (0.00,0.03)	0.00 (0.00,0.02)	0.00 (0.00,0.00)	**REML ITS** **﻿REML MA**

The top row of the label indicates the ITS analysis methods, bottom row indicates the meta-analysis method, e.g., OLS Fixed is OLS ITS analysis and fixed effect meta-analysis. For example, the median between-study variance for immediate level-change yielded by REML ITS analysis with REML estimator 0.08 higher than that yielded by OLS ITS analysis with DL estimator (column 4, row 1)

*DL* DerSimonian and Laird, *HKSJ* Hartung-Knapp/Sidik-Jonkman, *ITS* interrupted time series, *IQR* interquartile range, *MA *meta-analysis, *OLS* ordinary least squares, *REML* restricted maximum likelihood, *WT* Wald-type

### Sensitivity analysis

In our sensitivity analysis, we excluded 16 ITS from 5 meta-analyses. The results of the sensitivity analysis did not differ substantively from the primary analyses. Details of the differences between the meta-analyses in the primary analysis and the sensitivity analysis are presented in Additional file 1: Table S3; summary results are provided in Additional file 1: Appendix 3.

### Repository of ITS data

The ITS datasets analysed in this study, for which the authors gave consent (for 16 of 17 meta-analyses) are provided in an online repository: 10.26180/21280791 [51]. For each dataset, we describe the intervention and outcome examined, any changes made to the original meta-analysis to suit our purposes, and indicate for each ITS, the time, interval of time, time of interruption, segment in segmented linear regression model, the observation and its outcome type, and whether the ITS study was excluded from our sensitivity analysis.

## Discussion

### Summary and discussion of key findings

To our knowledge, no previous studies have empirically examined implications of different statistical methods for ITS analysis and meta-analysis using real-world ITS data. We created a repository of 17 meta-analyses including 282 ITS studies. We reanalysed each ITS study using two ITS analysis methods, and then meta-analysed the level-change and slope-change effects using five meta-analysis methods. We compared the impact of using different statistical methods on the meta-analytic level- and slope-change effect estimates, standard errors, confidence intervals and *p*-values. The results of our empirical study provide insight into the behaviour of ITS analysis and meta-analysis methods when applied to real-world ITS data.

When fixed-effect meta-analysis was used, our results indicated that there may be differences in the estimated meta-analytic effect for a given meta-analysis. However, the immediate level-change effect estimates yielded by REML ITS analysis were only slightly larger, on average, compared with OLS, which was likely driven by a single meta-analysis result. In addition, while on average we found unimportant differences in the estimated standard errors of the meta-analytic effects between the ITS analysis methods, for a given meta-analysis, there could be important differences. Estimated standard errors of the fixed-effect meta-analytic effects between the ITS methods have been shown (via numerical simulation [31]) to importantly differ for short series or where the underlying autocorrelation tends to be larger (i.e., at least 0.4). In the present dataset, some of the series were short and had autocorrelation greater than 0.4 potentially explaining the differences.

When random-effects meta-analysis was used, we found that on average the estimates of the random-effects meta-analytic effects of level- and slope-changes and their standard errors, were not impacted by the choice of random-effects meta-analysis method, irrespective of the ITS analysis method used. As expected, however, the standard errors were substantially larger compared with a fixed-effect model, due to the between-ITS variance (which was commonly estimated as greater than zero) being accounted for in the random-effects model. Furthermore, we found that the between-study variance estimates did not systematically differ by ITS analysis method or between-study variance estimator; which has been observed in other studies [29, 31]. However, the confidence interval method was shown to impact the confidence interval widths and statistical significance of the meta-analytic level-changes. This was primarily driven by the use of the t-distribution in the calculation of the confidence interval limits when using the HKSJ confidence interval method, rather than the small sample adjustment to the meta-analytic standard error. The consequence of wider confidence intervals and more conservative *p*-values when using HKSJ compared to WT, is that the conclusions drawn from the meta-analysis may differ.

### Strengths and limitations

Our study has several strengths. We examined ten statistical analysis combinations, which we compared using the metrics typically important to researchers undertaking meta-analysis, i.e., the meta-analytic point estimates, between-study variance estimates, confidence intervals, and *p*-values. Furthermore, the included systematic reviews and meta-analyses varied by the types of interruptions examined, the outcomes, the number of included studies per meta-analysis, and the number of datapoints per ITS study. The repository of ITS datasets has been made publicly available in an online repository, facilitating future methodological and statistical research.

Our study has several limitations. We were able to obtain raw ITS data from 17 of the 40 reviews included in our methodological review. While a small number of datasets is common in empirical methodological research [46, 54–57], this hinders examination of factors that may modify how the methods compare (e.g., the number of studies per meta-analysis). Furthermore, with a small number of datasets, outliers have more influence and parameters (such as the limits of agreement) are estimated with more uncertainty. In addition, we made several assumptions when analysing the ITS studies which may not hold (e.g., assuming count outcomes were continuous); we did not adjust for potential confounders (that may have been adjusted for in the original analysis); and, we fitted a segmented linear regression model with lag-1 autocorrelation (which may have differed to the original analysis and may not have provided the best fit). However, for reasons of feasibility and our interest in comparing the statistical methods and not in addressing the research question examined in the original meta-analysis, meant that we did not assess the fit or modify the models for the 282 included ITS studies.

### Implications for practice

We have demonstrated that the statistical methods for ITS analysis and meta-analysis do not, on average, impact the meta-analytic level- and slope-change effect estimates, their standard errors or the between-study variance estimates. However, across ITS analysis methods, for any given meta-analysis, there could be small to moderate differences in meta-analytic effect estimates, and important differences in the meta-analytic standard errors. Furthermore, the confidence intervals and *p*-values may be impacted. This demonstrates that in practice the statistical methods choices we have investigated may materially impact the results and conclusions, and the methods should therefore not be considered interchangeable. In this circumstance, numerical simulation studies provide the best evidence as to which methods are optimal under different scenarios (e.g., meta-analyses including short series), and we refer readers to our companion numerical simulation study for recommendations [31]. Furthermore, given the choice of methods can impact the results, it is even more important that the specific ITS analysis and meta-analysis methods used are reported. A systematic review examining the statistical methods used in meta-analysis of ITS studies found that while the ITS estimation method could almost always be determined (in 95% of reviews), if and how autocorrelation was accounted for could only be determined in 59% of reviews, and the between-study variance estimator and confidence interval method for the combined effect could only be determined in 60% and 57% of meta-analyses examined in the systematic review, respectively [26]. Hence much needs to be improved in reporting ITS studies.

### Implications for future research

Our ITS data repository may be expanded, facilitating other methodological and statistical research. Our research could be extended to examine the impact of ITS methods for analysing other outcome types, particularly count outcomes, due to their frequent use in ITS studies. Furthermore, our examinations could be expanded to accommodate increasing autocorrelation lags and seasonal patterns. In addition, we have not examined the impacts of the statistical methods on meta-analytic effect prediction intervals, which provide a predicted range for the true interruption effect in an individual study, and are a critical tool for decision-making [58]. Understanding the implications of statistical method choice on the prediction intervals is an important next step given the known impact of the ITS analysis methods on the estimation of between-study variance [31].

### Conclusions

We found on *average* minimal impact of statistical method choice on the meta-analysis effect estimates, their standard errors or the between-study variance estimates. However, across ITS analysis methods, for any given meta-analysis, there could be small to moderate differences in meta-analytic effect estimates, and important differences in the meta-analytic standard errors. Furthermore, we found that confidence intervals and *p*-values could vary according to the choice of statistical method. These differences may materially impact the results and conclusions and suggest that the statistical methods are not interchangeable in practice.

### Supplementary Information

**Additional file 1: Appendix 1.** Example ITS study and descriptions of meta-analysis modifications. **Appendix 2.** Additional results tables and figures. **Appendix 3.** Sensitivity analysis results. **Appendix 4.** Reviews that contributed data.

## Data Availability

The datasets and code used in this empirical study are available in the Monash Bridges repository, 10.26180/2128079151.

## Abbreviations

AR(1): Lag-1 autocorrelation
ITS: Interrupted Time Series
OLS: Ordinary Least Squares
REML: REstricted Maximum Likelihood
PW: Prais-Winsten
DL: DerSimonian and Laird between-study variance estimator
WT: Wald-type confidence interval method
HKSJ: Hartung-Knapp/Sidik-Jonkman confidence interval method
