# Supplementary material for: Comparison of statistical methods used to meta-analyse results from interrupted time series studies: an empirical study
Source: BMC Med Res Methodol. 2024 Feb 10;24:31. doi: 10.1186/s12874-024-02147-z (PMC10858609; doi:10.1186/s12874-024-02147-z)
Supplement: Supplementary file 1 — Additional file 1: Appendix 1. Example ITS study and descriptions of meta-analysis modifications. Appendix 2. Additional results tables and figures. Appendix 3. Sensitivity analysis results. Appendix 4. Reviews that contributed data. [file 12874_2024_2147_MOESM1_ESM.docx]

# Supplementary file 1: Supplementary tables and figures for Comparison of statistical methods used to meta-analyse results from interrupted time series studies: an empirical study

Appendix 1 – Example ITS study and descriptions of meta-analysis modifications

Appendix 2 – Additional results tables and figures

Appendix 3 – Sensitivity analysis results

Appendix 4 – Reviews that contributed data

## Appendix 1 – Example ITS study and descriptions of meta-analysis modifications

### Appendix Figure S1 Example of interrupted time series data

| 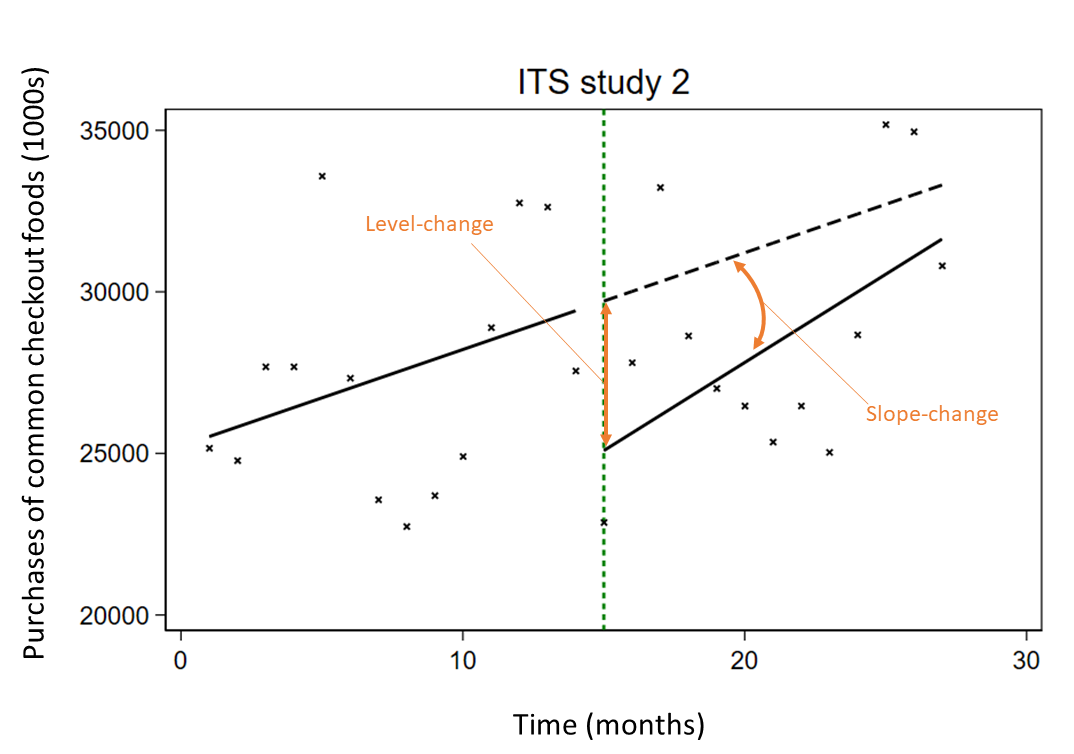 |
| --- |
| Appendix Figure S1. Example of interrupted time series data plotted for Study 2; shows the purchased of common checkout foods over time (months) before (left of the vertical green dashed line) and after (right) the introduction of supermarket policies for checkout food items1. The crosses represent data points, the solid black lines represent the pre- and post-interruption trend lines and the dashed black line represents the counterfactual trend line. |

### Appendix Table S1. Description and justification of the modifications to the original meta-analysis and our assumptions/modifications to the included ITS studies

| **Modification reference number**  **A indicates meta-analysis modification,**  **B indicates ITS study modification** | **Modification topic** | **Original analysis** | **Modification description** | **Justification** |
| --- | --- | --- | --- | --- |
| A1 | ITS definition | The authors of the original studies may have used a different definition of an ITS; for example, the series may not have had any requirements on the minimum number of data points to be considered an ITS. | Series that did not meet the series inclusion criteria (Section 2.1) were excluded from the meta-analysis:   1. a series of datapoints collected on a group of individuals 2. the series contained at least three datapoints before and after an interruption with a clearly defined timepoint | There is a lack of consensus on the minimum number of data points required for appropriate statistical analysis, however, we chose to follow the EPOC guidelines, which state a minimum number of three datapoints before and after an interruption are required. |
| A2 | Control series | The original meta-analyses may have included control series in their statistical analysis (e.g., an effect estimate may have been adjusted for any difference observed in a control series). | We included analysis of interrupted series only. We did not incorporate any information from control series (i.e. if control series were provided, they were not included in the meta-analysis in any way). | Statistical methods for the inclusion of control series in a meta-analysis are beyond the scope of this study. |
| A3 | Multiple units of time within a meta-analysis | The original study included ITS studies have different data points aggregated at different units of time (e.g., some ITS with weekly datapoints and other with monthly datapoints). | We included series using the most common aggregation period in the meta-analysis (i.e., if 5 ITS had weekly datapoints and 6 had monthly datapoints, we would include only the 6 with monthly datapoints). | We ensured that the same unit of time between datapoints was consistent within each meta-analysis, to ensure comparability of all effect estimates being meta-analysed. Conversion to different time unit aggregations was not considered as this may present more complications than it resolves. |
| A4 | Outcome types – observations at or near upper or lower limits | The original study may include ITS with observed datapoints at or near an upper or lower limit, such as zeros (or 1 in the case of proportions). | We excluded ITS studies that had a high proportion of zeros (or ones) were excluded from the analysis.  All studies with zeros were discussed by the authors. Where the review team could not determine whether the proportion of zeros in a particular study would violate the assumptions of the continuous outcome model, we included the series in the primary but excluded it from the sensitivity analysis. | The continuous outcome model in Section 2.4.1 cannot be reasonably applied to series with an excess of observations at an upper or lower cap (i.e., the assumption of normally distributed data would be violated).  The discussion among the authors concluded that an “excess” of zeros was defined as greater than 40% of the total number of datapoints, while series with greater than 30% of the total number of datapoints would be excluded in the sensitivity analysis. |
| B5 | Multiple study designs within a meta-analysis | The original study included multiple study design in the meta-analysis (e.g., combining the effect estimates extraction from RCT and ITS studies). | Only studies with ITS designs were included, i.e. we excluded studies with other designs, such as RCTs. | Our interest was in ITS studies only. |
| A6 | Subgroup analysis | The original study conducted a subgroup meta-analysis. | We included all studies, from all subgroups in the same meta-analysis. No subgroup analysis was performed. | Our interest was not to reproduce the original meta-analyses. |
| A7 | Network meta-analysis | The original study examined multiple intervention comparisons in a network meta-analysis. | We selected only ITS studies that contributed to a single direct pairwise comparison. The pairwise comparison with the most contributing ITS studies was chosen. | We focus on pairwise meta-analysis method; network meta-analysis methods are beyond the scope of this study. |
| B1 | Adjustments for confounding | The original study included additional variables in their model (in addition to the core ITS covariates described in section 2.4.1). For example, models adjusted for population growth or population demographics. | No additional covariates were added to the models described in section 2.4.1. Population adjustments were only made if it was possible to calculate rates. | Our interest was not to reproduce the original meta-analysis. |
| B2 | Outcome types – outcomes other than continuous | The original study included ITS with data that was not continuous (e.g., counts, binary, rate, proportion). | We included all ITS studies, irrespective of their outcome type. The outcomes were treated as continuous outcomes in our analysis (according to the model described in section 2.4.1). | Outcome types that are not continuous, when aggregated, may be modelled using the continuous outcome model described in Section 2.4.1 without violating the underlying assumptions (e.g., normally distributed error term). |
| B3 | Transition periods | The original study examined an interruption that had an anticipated delay, or transition period (e.g., the interruption was rolled out over a period of time). Thus the effect estimates of interest were calculated some period of time after the start of the interruption. The original analysis may or may not have separately modelled the transition and post-interruption periods. | For series that we were aware the interruptions spanned multiple time points (i.e., the transition period), we included an additional segment for the transition period and calculated the immediate level-change, defined at the difference between the outcome observed and the projected outcome by the pre-interruption period trend at the first time point of the post-interruption period (after the transition period). | Our interest was in the immediate-level-change and slope-change when comparing the pre-interruption period and post-interruption period. As such, we modelled the transition period, however, in when calculating the interruption effects of interest, we ignored the transition period segment.  This ITS study modification was defined in our protocol, however no ITS studies included required this modification. |
| B4 | Multiple interruptions | The original study examined multiple interruptions (e.g., an intervention with two phases to its implementation will have two defined interruption points, and three segments). | We included only the segments before and after the first interruption. | Our interest was in the regression parameters yielded by the model described in Section 2.4.1. |
| B5 | Single interruption at multiple time points in a single series | The original study may have examined a single interruption that occurred repeatedly over time (i.e. a single time series with repeat interruptions, as opposed to several series each with a single interruption; a meta-analysis may be conducted across time as opposed to series, e.g., heavy rainfall events Phung 2017^2^). | The single series was divided into multiple series. The point of division between two interruptions was at the midpoint. | Equal division of the datapoints between two interruptions was conducted to ensure that there were no datapoints contributing to multiple “interrupted time series” (i.e., each datapoint contributed only once in the meta-analysis). |

## Appendix 2 – Additional results tables and figures

### Appendix Figure S2. Plots of the standardised effect estimates and confidence intervals for each meta-analysis

| 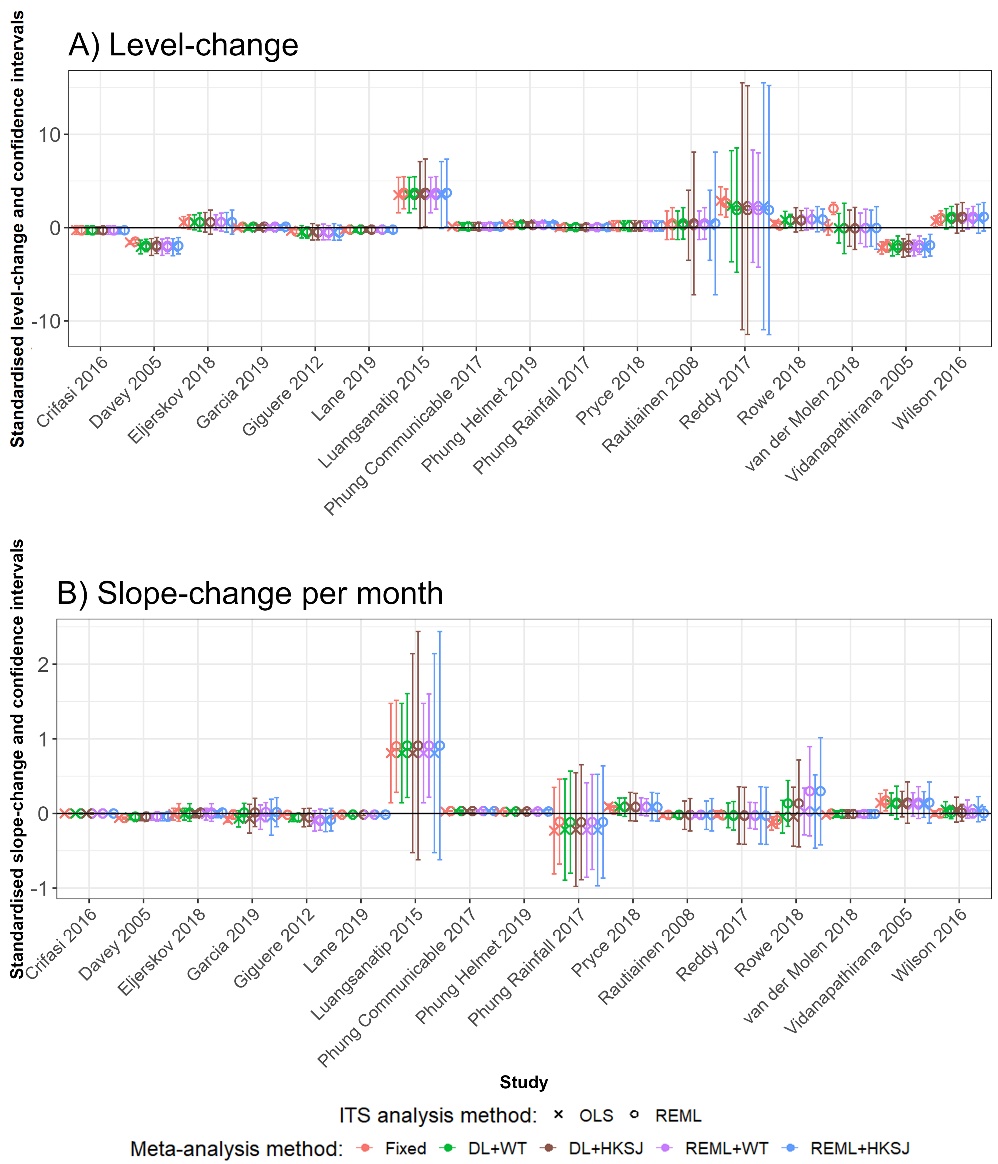 | Appendix Figure S2. Plots of the standardised effect estimates and confidence intervals for each meta-analysis (x-axis) for level-change (A) and slope-change per month (B) when the included ITS studies were analysed with OLS (crosses) and REML (open circles), and when fixed-effect (red), DL+WT (green) and REML+HKSJ (blue) meta-analysis methods were used.  Standardised effects were obtained through division by the root mean square error (RMSE) from the OLS analysis.  DL, DerSimonian and Laird. HKSJ, Hartung-Knapp / Sidik-Jonkman. ITS, interrupted time series. OLS, ordinary least squares. REML, restricted maximum likelihood. WT, Wald-type. |
| --- | --- |

### Appendix Figure S3. Pairwise comparison of confidence intervals yielded by combinations of ITS analysis (OLS or REML) and meta-analysis methods (DL+WT, DL+HKSJ, REML+WT or REML+HKSJ).

| 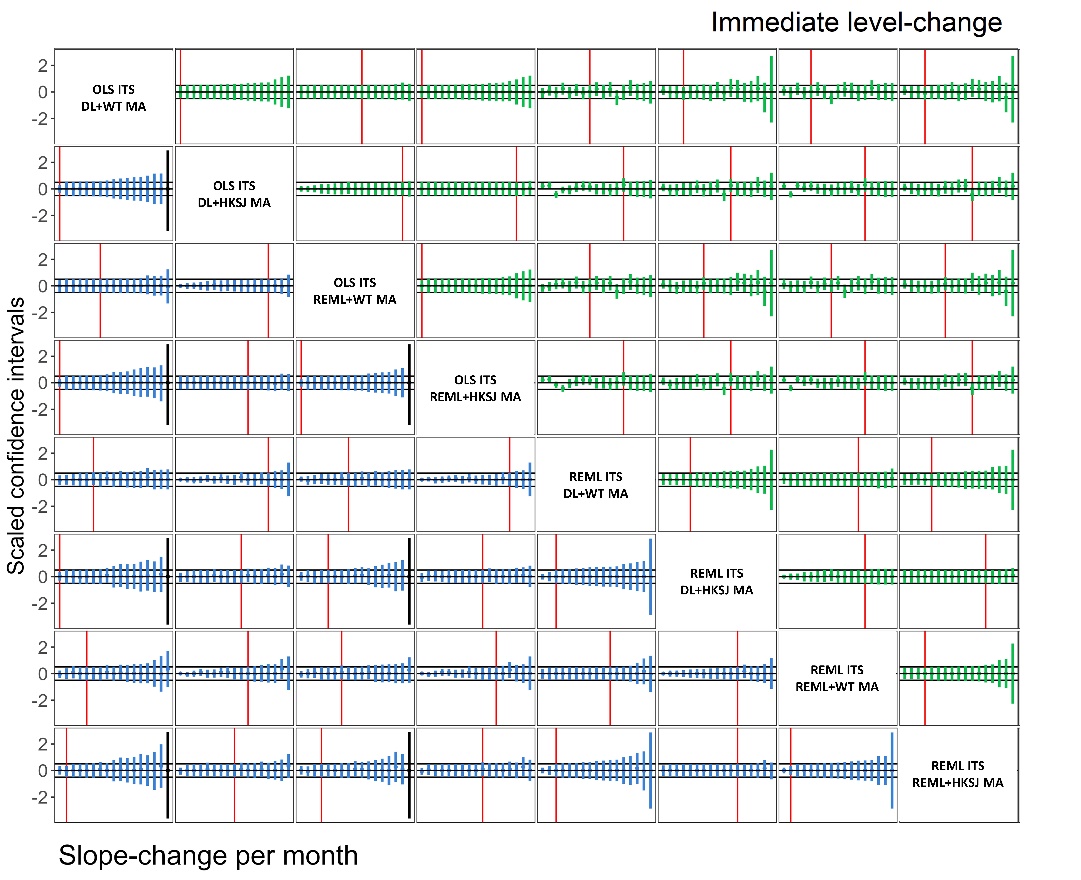 |
| --- |
| Appendix Figure S3. Pairwise comparison of confidence intervals yielded by combinations of ITS analysis (OLS or REML) and meta-analysis methods (DL+WT, DL+HKSJ, REML+WT or REML+HKSJ). Each plot contains the 17 meta-analyses’ absolute difference in meta-analytic effect estimates and scaled relative confidence intervals, ranked in order of scaled relative confidence interval width. The top triangle (green points) presents the immediate level-change, while the bottom triangle (blue points) presents the slope-change per month. The scaled relative confidence interval widths for the level-change were calculated as column method confidence interval width divided by row method confidence interval width (and row method / column method for slope-change per month), scaled such that the row method (column method in the case of slope-change per month) spans -0.5 to 0.5 (indicated by the horizontal grey lines, which form the ‘reference rectangle’). Confidence intervals entirely within the reference rectangle (i.e., between the horizontal grey lines) have smaller confidence intervals than the comparison (left of the vertical red line), while the confidence intervals extending beyond the reference rectangle have larger confidence intervals than the comparison (right of the vertical red line). The black confidence intervals indicate where one or both of the confidence limits were beyond the limits y-axis scale.  For example, in column 3, row 1, we can see that the level-change confidence intervals yielded by OLS ITS analysis with DL+WT meta-analysis are of the same/similar width to those yielded by OLS ITS analysis with REML+WT meta-analysis. Whereas in column 1, row 8, we can see that using REML ITS analysis with REML+HKSJ meta-analysis yields a couple of narrower slope-change per month confidence intervals or similar confidence intervals, but mostly wider confidence intervals, than OLS ITS analysis with DL+WT meta-analysis.  DL, DerSimonian and Laird. HKSJ, Hartung-Knapp / Sidik-Jonkman. ITS, interrupted time series. MA, meta-analysis. OLS, ordinary least squares. REML, restricted maximum likelihood. WT, Wald-type. |

### Appendix Table S2. Frequencies of agreement in statistical significance of p-values (categorised as p<0.05 or p$\geq$0.05) for pairwise comparisons of the meta-analytic immediate level-change (top triangle) and slope-change per month (bottom triangle) for combinations of ITS analysis and meta-analysis methods. (n = 17)

|  |  | **Level-change** | | | | | | | | | | | | | | | | | | | |
| --- | --- | --- | --- | --- | --- | --- | --- | --- | --- | --- | --- | --- | --- | --- | --- | --- | --- | --- | --- | --- | --- |
| **Slope-change per month** |  | p<0.05 | p≥0.05 | p<0.05 | p≥0.05 | p<0.05 | p≥0.05 | p<0.05 | p≥0.05 | p<0.05 | p≥0.05 | p<0.05 | p≥0.05 | p<0.05 | p≥0.05 | p<0.05 | p≥0.05 | p<0.05 | p≥0.05 | p<0.05 | p≥0.05 |
|  | p≥0.05 | **OLS ITS**  **Fixed MA** | | 0 | 8 | 0 | 8 | 0 | 8 | 0 | 8 | 1 | 7 | 0 | 8 | 0 | 8 | 0 | 8 | 0 | 8 |
|  | p<0.05 |  |  | 4 | 5 | 3 | 6 | 4 | 5 | 3 | 6 | 7 | 2 | 6 | 3 | 4 | 5 | 5 | 4 | 3 | 6 |
|  | p≥0.05 | 0 | 7 | **OLS ITS**  **DL+WT MA** | | 0 | 13 | 0 | 13 | 0 | 13 | 4 | 9 | 2 | 11 | 0 | 13 | 1 | 12 | 0 | 13 |
|  | p<0.05 | 6 | 4 |  |  | 3 | 1 | 4 | 0 | 3 | 1 | 4 | 0 | 4 | 0 | 4 | 0 | 4 | 0 | 3 | 1 |
|  | p≥0.05 | 0 | 7 | 0 | 11 | **OLS ITS**  **DL+HKSJ MA** | | 1 | 13 | 0 | 14 | 5 | 9 | 3 | 11 | 1 | 13 | 2 | 12 | 1 | 13 |
|  | p<0.05 | 4 | 6 | 4 | 2 |  |  | 3 | 0 | 3 | 0 | 3 | 0 | 3 | 0 | 3 | 0 | 3 | 0 | 2 | 1 |
|  | p≥0.05 | 0 | 7 | 0 | 11 | 1 | 12 | **OLS ITS**  **REML+WT MA** | | 0 | 13 | 4 | 9 | 2 | 11 | 0 | 13 | 1 | 12 | 0 | 13 |
|  | p<0.05 | 4 | 6 | 4 | 2 | 3 | 1 |  |  | 3 | 1 | 4 | 0 | 4 | 0 | 4 | 0 | 4 | 0 | 3 | 1 |
|  | p≥0.05 | 0 | 7 | 0 | 11 | 0 | 13 | 0 | 13 | **OLS ITS**  **REML+HKSJ MA** | | 5 | 9 | 3 | 11 | 1 | 13 | 2 | 12 | 1 | 13 |
|  | p<0.05 | 3 | 7 | 3 | 3 | 3 | 1 | 3 | 1 |  |  | 3 | 0 | 3 | 0 | 3 | 0 | 3 | 0 | 2 | 1 |
|  | p≥0.05 | 0 | 7 | 2 | 9 | 4 | 9 | 4 | 9 | 5 | 9 | **REML ITS**  **Fixed MA** | | 1 | 8 | 0 | 9 | 0 | 9 | 0 | 9 |
|  | p<0.05 | 7 | 3 | 5 | 1 | 3 | 1 | 3 | 1 | 2 | 1 |  |  | 5 | 3 | 4 | 4 | 5 | 3 | 3 | 5 |
|  | p≥0.05 | 0 | 7 | 0 | 11 | 1 | 12 | 1 | 12 | 2 | 12 | 0 | 10 | **REML ITS**  **DL+WT MA** | | 0 | 11 | 0 | 11 | 0 | 11 |
|  | p<0.05 | 4 | 6 | 4 | 2 | 3 | 1 | 3 | 1 | 2 | 1 | 4 | 3 |  |  | 4 | 2 | 5 | 1 | 3 | 3 |
|  | p≥0.05 | 0 | 7 | 0 | 11 | 0 | 13 | 0 | 13 | 0 | 14 | 1 | 9 | 1 | 12 | **REML ITS**  **DL+HKSJ MA** | | 1 | 12 | 0 | 13 |
|  | p<0.05 | 3 | 7 | 3 | 3 | 3 | 1 | 3 | 1 | 3 | 0 | 2 | 5 | 2 | 2 |  |  | 4 | 0 | 3 | 1 |
|  | p≥0.05 | 0 | 7 | 0 | 11 | 1 | 12 | 0 | 13 | 1 | 13 | 0 | 10 | 0 | 13 | 1 | 13 | **REML ITS**  **REML+WT MA** | | 0 | 12 |
|  | p<0.05 | 3 | 7 | 3 | 3 | 2 | 2 | 3 | 1 | 2 | 1 | 3 | 4 | 3 | 1 | 2 | 1 |  |  | 3 | 2 |
|  | p≥0.05 | 0 | 7 | 0 | 11 | 0 | 13 | 0 | 13 | 0 | 14 | 1 | 9 | 1 | 12 | 0 | 14 | 1 | 13 | **REML ITS**  **REML+HKSJ MA** | |
|  | p<0.05 | 3 | 7 | 3 | 3 | 3 | 1 | 3 | 1 | 3 | 0 | 2 | 5 | 2 | 2 | 3 | 0 | 2 | 1 |  |  |
| DL, DerSimonian and Laird. HKSJ, Hartung-Knapp / Sidik-Jonkman. ITS, interrupted time series. MA, meta-analysis. OLS, ordinary least squares. REML, restricted maximum likelihood. WT, Wald-type. | | | | | | | | | | | | | | | | | | | | | |

## Appendix 3 – Sensitivity analysis results

### Appendix Table S3. Differences in the number of ITS studies included in the primary analysis and the sensitivity analysis.

| **Citation** | **Review** | **Primary analysis** | **Sensitivity analysis** |
| --- | --- | --- | --- |
| Crifasi CK, Pollack KM, Webster DW. Effects of state-level policy changes on homicide and nonfatal shootings of law enforcement officers. Inj Prev. 2016 Aug;22(4):274-8. doi: 10.1136/injuryprev-2015-041825. Epub 2015 Dec 30. PMID: 26718550. | Crifasi 2016 | 24 ITS studies were provided. For our primary analysis, we excluded 12 because a high proportion of datapoints were 0 (n = 12). | We excluded an additional five ITS studies because of a high proportion of zero observations (n = 7). |
| Davey P, Marwick CA, Scott CL, Charani E, McNeil K, Brown E, Gould IM, Ramsay CR, Michie S. Interventions to improve antibiotic prescribing practices for hospital inpatients. Cochrane Database Syst Rev. 2017 Feb 9;2(2):CD003543. doi: 10.1002/14651858.CD003543.pub4. PMID: 28178770; PMCID: PMC6464541. | Davey 2017 | 88 of the 91 ITS studies examined in the original meta-analysis were provided by the authors. We extracted the raw ITS data from 1 of the 3 ITS studies not provided (n = 89). For our primary analysis, we excluded 32 ITS studies because they examined a different interruption type (we limited our analysis to Enablement interruptions) and excluded 11 ITS studies that did not use the most common unit of time (i.e., the most common interval was monthly while the excluded studies used quarterly/2 weekly intervals) (n = 46). | We excluded an additional five ITS studies because a high proportion of datapoints were 0 or 100 (n = 41). |
| Garcia-Elorrio E, Rowe SY, Teijeiro ME, Ciapponi A, Rowe AK. The effectiveness of the quality improvement collaborative strategy in low- and middle-income countries: A systematic review and meta-analysis. PLoS One. 2019 Oct 3;14(10):e0221919. doi: 10.1371/journal.pone.0221919. PMID: 31581197; PMCID: PMC6776335. | Garcia-Elorrio 2019 | 14 ITS studies were provided. For our primary analysis, we excluded 1 because a high proportion of datapoints were 0 (n = 13). | We excluded an additional two ITS because a high proportion of datapoints were at or near 0 or 100 (n = 11) |
| Pryce J, Choi L, Richardson M, Malone D. Insecticide space spraying for preventing malaria transmission. Cochrane Database Syst Rev. 2018 Nov 2;11(11):CD012689. doi: 10.1002/14651858.CD012689.pub2. PMID: 30388303; PMCID: PMC6516806. | Pryce 2018 | Four ITS studies were provided. All four ITS studies were analysed in the primary analysis. | We excluded an additional one ITS study because a high proportion of datapoints were 0 (n = 3). |
| Rowe AK, Rowe SY, Peters DH, Holloway KA, Chalker J, Ross-Degnan D. Effectiveness of strategies to improve health-care provider practices in low-income and middle-income countries: a systematic review. Lancet Glob Health. 2018 Nov;6(11):e1163-e1175. doi: 10.1016/S2214-109X(18)30398-X. Epub 2018 Oct 8. PMID: 30309799; PMCID: PMC6185992. | Rowe 2018 | Multiple study designs were included in the original meta-analysis. We included only the 15 ITS studies. | We excluded an additional three ITS because a high proportion of datapoints were 0 or 100 (n = 12). |

### Appendix Table S4. Comparing the primary vs sensitivity analysis results for the mean difference of effect estimates and 95% limits of agreement for the meta-analytic immediate level-change (top triangle, difference calculated as column method - row method) and slope-change per month (bottom triangle, difference calculated as row method - column method) (n = 17)

|  | **Level-change** | | | | | |
| --- | --- | --- | --- | --- | --- | --- |
| **Slope-change per month** | **OLS ITS**  **Fixed MA** | -0.04 (-0.48,0.40)  vs  -0.06 (-0.47,0.34) | -0.03 (-0.50,0.43)  vs  -0.07 (-0.48,0.35) | 0.15 (-0.85,1.15)  vs  0.15 (-0.84,1.15) | -0.01 (-0.65,0.63)  vs  -0.03 (-0.65,0.59) | 0.00 (-0.66,0.66)  vs  -0.03 (-0.65,0.59) |
|  | 0.01 (-0.05,0.06)  vs  0.01 (-0.04,0.06) | **OLS ITS**  **DL MA** | 0.00 (-0.04,0.04)  vs  0.00 (-0.02,0.01) | 0.19 (-0.90,1.27)  vs  0.22 (-0.82,1.25) | 0.03 (-0.30,0.36)  vs  0.03 (-0.29,0.35) | 0.04 (-0.29,0.36)  vs  0.03 (-0.28,0.35) |
|  | 0.01 (-0.08,0.10)  vs  0.02 (-0.12,0.15) | 0.00 (-0.04,0.04)  vs  0.01 (-0.08,0.10) | **OLS ITS**  **REML MA** | 0.19 (-0.92,1.29)  vs  0.22 (-0.82,1.26) | 0.03 (-0.31,0.37)  vs  0.04 (-0.28,0.36) | 0.03 (-0.29,0.36)  vs  0.04 (-0.28,0.35) |
|  | 0.02 (-0.06,0.10)  vs  0.02 (-0.06,0.10) | 0.01 (-0.07,0.10)  vs  0.01 (-0.07,0.09) | 0.01 (-0.10,0.12)  vs  0.00 (-0.14,0.14) | **REML ITS**  **Fixed MA** | -0.16 (-1.28,0.96)  vs  -0.18 (-1.27,0.90) | -0.15 (-1.27,0.96)  vs  -0.18 (-1.25,0.88) |
|  | 0.04 (-0.11,0.18)  vs  0.04 (-0.10,0.18) | 0.03 (-0.08,0.14)  vs  0.03 (-0.08,0.15) | 0.03 (-0.06,0.11)  vs  0.02 (-0.06,0.11) | 0.01 (-0.10,0.13)  vs  0.02 (-0.10,0.14) | **REML ITS**  **DL MA** | 0.01 (-0.05,0.07)  vs  0.00 (-0.03,0.03) |
|  | 0.04 (-0.17,0.26)  vs  0.06 (-0.18,0.30) | 0.04 (-0.14,0.21)  vs  0.05 (-0.16,0.26) | 0.03 (-0.11,0.18)  vs  0.04 (-0.10,0.18) | 0.02 (-0.17,0.21)  vs  0.04 (-0.18,0.26) | 0.01 (-0.07,0.09)  vs  0.02 (-0.09,0.12) | **REML ITS**  **REML MA** |
| The primary analysis included 17 meta-analyses with 282 included ITS studies. The sensitivity analysis included 17 meta-analyses with 266 included ITS studies, with the 16 ITS studies being removed from 5 meta-analyses because the author team could not reach consensus on whether to include them in the primary analysis (e.g., underlying model assumption may be violated).  Interpreting the table – example 1: the mean meta-analytic level-change yielded by REML ITS analysis with fixed effect meta-analysis was 0.15 higher than that yielded by OLS ITS analysis with fixed effect meta-analysis in both the primary analysis and the sensitivity analysis (column 4, row 1). Example 2: in both the primary and sensitivity analyses, the mean meta-analytic slope-change per month yielded by REML ITS analysis with fixed effect meta-analysis was 0.02 higher than that yielded by OLS ITS analysis with fixed effect meta-analysis (column 1, row 4).  DL, DerSimonian and Laird. HKSJ, Hartung-Knapp / Sidik-Jonkman. ITS, interrupted time series. MA, meta-analysis. OLS, ordinary least squares. REML, restricted maximum likelihood. WT, Wald-type. | | | | | | |

### Appendix Table S5. Comparing the primary vs sensitivity analysis results for the mean ratio of standard errors and 95% limits of agreement for the meta-analytic immediate level-change (top triangle, ratio calculated as column method / row method) and slope-change per month (bottom triangle, ratio calculated as row method / column method) (n = 17).

|  | **Level-change** | | | | | | | | | |
| --- | --- | --- | --- | --- | --- | --- | --- | --- | --- | --- |
| **Slope-change per month** | **OLS ITS**  **Fixed MA** | 1.70 (0.59,4.94)  vs  1.66 (0.58,4.82) | 1.60 (0.37,6.90)  vs  1.53 (0.37,6.35) | 1.78 (0.55,5.73)  vs  1.72 (0.56,5.33) | 1.61 (0.37,6.99)  vs  1.54 (0.37,6.39) | 1.06 (0.71,1.57)  vs  1.08 (0.74,1.58) | 1.75 (0.63,4.85)  vs  1.78 (0.67,4.72) | 1.61 (0.43,6.02)  vs  1.58 (0.43,5.83) | 1.80 (0.61,5.32)  vs  1.81 (0.66,4.98) | 1.62 (0.43,6.12)  vs  1.59 (0.43,5.89) |
|  | 2.10 (0.61,7.22)  vs  2.03 (0.61,6.80) | **OLS ITS**  **DL+WT MA** | 0.94 (0.54,1.64)  vs  0.92 (0.54,1.57) | 1.04 (0.88,1.24)  vs  1.03 (0.88,1.21) | 0.94 (0.53,1.67)  vs  0.92 (0.54,1.58) | 0.62 (0.20,1.95)  vs  0.65 (0.21,2.05) | 1.03 (0.68,1.55)  vs  1.07 (0.71,1.61) | 0.95 (0.61,1.46)  vs  0.95 (0.60,1.50) | 1.06 (0.79,1.41)  vs  1.09 (0.80,1.47) | 0.95 (0.61,1.48)  vs  0.95 (0.60,1.51) |
|  | 2.29 (0.42,12.57)  vs  2.22 (0.40,12.17) | 1.09 (0.57,2.09)  vs  1.09 (0.56,2.11) | **OLS ITS**  **DL+HKSJ MA** | 1.11 (0.68,1.82)  vs  1.12 (0.69,1.83) | 1.00 (0.98,1.02)  vs  1.00 (0.99,1.01) | 0.66 (0.14,3.11)  vs  0.71 (0.16,3.15) | 1.09 (0.51,2.35)  vs  1.16 (0.57,2.37) | 1.01 (0.62,1.63)  vs  1.03 (0.66,1.62) | 1.13 (0.60,2.11)  vs  1.18 (0.64,2.17) | 1.01 (0.63,1.63)  vs  1.04 (0.66,1.62) |
|  | 2.42 (0.51,11.52)  vs  2.36 (0.49,11.49) | 1.15 (0.70,1.90)  vs  1.16 (0.65,2.07) | 1.06 (0.71,1.58)  vs  1.06 (0.68,1.66) | **OLS ITS**  **REML+WT MA** | 0.90 (0.55,1.48)  vs  0.89 (0.55,1.45) | 0.59 (0.17,2.11)  vs  0.63 (0.19,2.11) | 0.98 (0.59,1.63)  vs  1.03 (0.65,1.63) | 0.91 (0.61,1.36)  vs  0.92 (0.59,1.43) | 1.01 (0.75,1.37)  vs  1.05 (0.78,1.41) | 0.91 (0.61,1.36)  vs  0.92 (0.59,1.44) |
|  | 2.40 (0.40,14.49)  vs  2.33 (0.38,14.33) | 1.14 (0.52,2.49)  vs  1.14 (0.50,2.60) | 1.05 (0.89,1.24)  vs  1.05 (0.83,1.32) | 0.99 (0.62,1.56)  vs  0.99 (0.62,1.55) | **OLS ITS**  **REML+HKSJ MA** | 0.66 (0.14,3.13)  vs  0.71 (0.16,3.16) | 1.09 (0.50,2.37)  vs  1.16 (0.57,2.37) | 1.00 (0.62,1.63)  vs  1.03 (0.66,1.62) | 1.12 (0.60,2.11)  vs  1.18 (0.64,2.17) | 1.01 (0.62,1.63)  vs  1.03 (0.66,1.62) |
|  | 1.20 (0.80,1.79)  vs  1.23 (0.82,1.84) | 0.57 (0.17,1.95)  vs  0.60 (0.18,2.08) | 0.52 (0.09,2.94)  vs  0.55 (0.10,3.20) | 0.49 (0.10,2.35)  vs  0.52 (0.10,2.66) | 0.50 (0.08,3.08)  vs  0.53 (0.08,3.43) | **REML ITS**  **Fixed MA** | 1.65 (0.56,4.90)  vs  1.64 (0.57,4.74) | 1.52 (0.38,6.15)  vs  1.46 (0.36,5.92) | 1.70 (0.53,5.45)  vs  1.67 (0.56,4.94) | 1.53 (0.37,6.28)  vs  1.47 (0.36,5.98) |
|  | 2.31 (0.78,6.82)  vs  2.26 (0.76,6.74) | 1.10 (0.72,1.68)  vs  1.11 (0.62,2.01) | 1.01 (0.42,2.40)  vs  1.02 (0.39,2.66) | 0.95 (0.49,1.87)  vs  0.96 (0.43,2.16) | 0.96 (0.37,2.50)  vs  0.97 (0.34,2.78) | 1.93 (0.66,5.62)  vs  1.84 (0.62,5.49) | **REML ITS**  **DL+WT MA** | 0.92 (0.56,1.51)  vs  0.89 (0.52,1.54) | 1.03 (0.78,1.37)  vs  1.02 (0.81,1.28) | 0.93 (0.56,1.54)  vs  0.89 (0.52,1.55) |
|  | 2.40 (0.46,12.52)  vs  2.39 (0.44,13.09) | 1.14 (0.53,2.46)  vs  1.18 (0.46,2.99) | 1.05 (0.71,1.55)  vs  1.08 (0.56,2.07) | 0.99 (0.57,1.70)  vs  1.01 (0.48,2.15) | 1.00 (0.69,1.46)  vs  1.03 (0.55,1.93) | 2.00 (0.37,10.96)  vs  1.95 (0.34,11.17) | 1.04 (0.46,2.34)  vs  1.06 (0.47,2.40) | **REML ITS DL+HKSJ MA** | 1.12 (0.78,1.59)  vs  1.14 (0.72,1.82) | 1.01 (0.98,1.03)  vs  1.00 (0.99,1.01) |
|  | 2.58 (0.58,11.57)  vs  2.65 (0.55,12.81) | 1.23 (0.55,2.73)  vs  1.30 (0.49,3.44) | 1.13 (0.50,2.53)  vs  1.19 (0.47,3.05) | 1.06 (0.58,1.96)  vs  1.12 (0.52,2.43) | 1.08 (0.48,2.44)  vs  1.14 (0.46,2.84) | 2.15 (0.47,9.88)  vs  2.16 (0.44,10.60) | 1.12 (0.60,2.08)  vs  1.17 (0.62,2.23) | 1.08 (0.57,2.02)  vs  1.11 (0.61,2.01) | **REML ITS**  **REML+WT MA** | 0.90 (0.63,1.29)  vs  0.88 (0.55,1.40) |
|  | 2.44 (0.42,14.23)  vs  2.48 (0.39,15.59) | 1.16 (0.46,2.92)  vs  1.22 (0.40,3.66) | 1.07 (0.63,1.80)  vs  1.12 (0.52,2.40) | 1.01 (0.53,1.93)  vs  1.05 (0.44,2.47) | 1.02 (0.64,1.63)  vs  1.06 (0.53,2.15) | 2.04 (0.33,12.60)  vs  2.01 (0.30,13.37) | 1.06 (0.42,2.67)  vs  1.09 (0.42,2.87) | 1.02 (0.84,1.24)  vs  1.03 (0.83,1.29) | 0.95 (0.51,1.75)  vs  0.93 (0.52,1.69) | **REML ITS**  **REML+HKSJ MA** |
| The primary analysis included 17 meta-analyses with 282 included ITS studies. The sensitivity analysis included 17 meta-analyses with 266 included ITS studies, with the 16 ITS studies being removed from 5 meta-analyses because the author team could not reach consensus on whether to include them in the primary analysis (e.g., underlying model assumption may be violated).  The top row of the label indicates the ITS analysis methods, bottom row indicates the meta-analysis method, e.g., OLS ITS Fixed MA indicates OLS ITS analysis and fixed-effect meta-analysis.  Interpreting the table – example 1: in the primary analysis, on average the standard error of the meta-analytic level-change yielded by REML ITS analysis with fixed-effect meta-analysis was 6% higher than the standard error yielded by OLS ITS analysis with fixed-effect meta-analysis, but was 8% higher in the sensitivity analysis (column 6, row 1). Example 2: in primary and sensitivity analyses, the standard error of the meta-analytic slope-change per month yielded by REML ITS analysis with fixed-effect meta-analysis was, on average, 20% higher than the standard error yielded by OLS ITS analysis with fixed effect meta-analysis, but was 23% higher in the sensitivity analysis (column 1, row 6).  DL, DerSimonian and Laird. HKSJ, Hartung-Knapp / Sidik-Jonkman. ITS, interrupted time series. MA, meta-analysis. OLS, ordinary least squares. REML, restricted maximum likelihood. WT, Wald-type. | | | | | | | | | | |

### Appendix Figure S4. Comparing the primary and sensitivity analysis scaled pairwise comparison of confidence intervals yielded by combinations of ITS analysis and meta-analysis methods

| 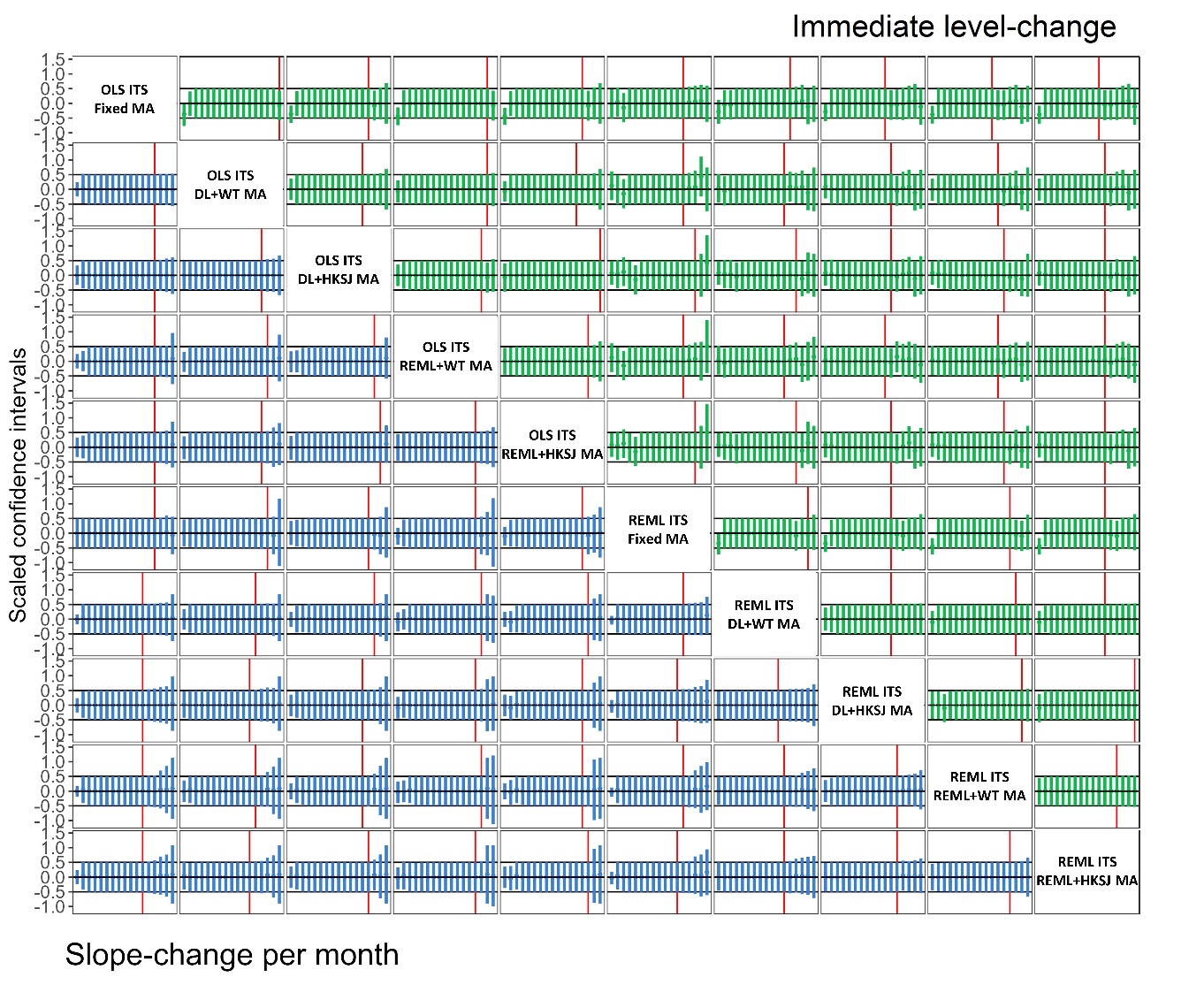 |
| --- |
| Figure S4. Pairwise comparison of sensitivity analysis confidence intervals vs primary analysis confidence intervals yielded by combinations of ITS analysis (OLS or REML) and meta-analysis methods (fixed, DL+WT, DL+HKSJ, REML+WT or REML+HKSJ). Each plot contains the 17 meta-analyses’ difference between the sensitivity analysis and primary analysis absolute differences in meta-analytic effect estimates and scaled relative confidence intervals for the sensitivity analysis vs the primary analysis, ranked in order of scaled relative confidence interval width. The top triangle (green points) presents the immediate level-change, while the bottom triangle (blue points) presents the slope-change per month. The scaled relative confidence interval widths for the level-change were calculated as column method confidence interval width divided by row method confidence interval width for the sensitivity analysis divided by the column method confidence interval width divided by row method confidence interval width (and row method / column method for slope-change per month), scaled such that the row method for the primary analysis (column method in the case of slope-change per month) spans -0.5 to 0.5 (indicated by the horizontal grey lines, which form the ‘reference rectangle’). Confidence intervals entirely within the reference rectangle (i.e., between the horizontal grey lines) have smaller confidence intervals than the comparison (left of the vertical red line), while the confidence intervals extending beyond the reference rectangle have larger confidence intervals than the comparison (right of the vertical red line).  For example, in column 4, row 2, we can see that the level-change pairwise confidence interval comparison between OLS ITS analysis with REML+WT meta-analysis vs OLS ITS analysis with DL+WT meta-analysis and in the sensitivity analysis were almost always the same as those in the primary analysis.  DL, DerSimonian and Laird. HKSJ, Hartung-Knapp / Sidik-Jonkman. ITS, interrupted time series. MA, meta-analysis. OLS, ordinary least squares. REML, restricted maximum likelihood. WT, Wald-type. |

### Appendix Table S6. Comparing the primary and sensitivity analysis results for the frequencies of agreement in statistical significance of p-values (categorised as p<0.05 or p$\geq$0.05) for pairwise comparisons of the meta-analytic immediate level-change (top triangle) and slope-change per month (bottom triangle) for combinations of ITS analysis and meta-analysis methods. (n = 17)

|  |  | **Level-change** | | | | | | | | | | | | | | | | | | | |
| --- | --- | --- | --- | --- | --- | --- | --- | --- | --- | --- | --- | --- | --- | --- | --- | --- | --- | --- | --- | --- | --- |
| **Slope-change per month** |  | p<0.05 | p≥0.05 | p<0.05 | p≥0.05 | p<0.05 | p≥0.05 | p<0.05 | p≥0.05 | p<0.05 | p≥0.05 | p<0.05 | p≥0.05 | p<0.05 | p≥0.05 | p<0.05 | p≥0.05 | p<0.05 | p≥0.05 | p<0.05 | p≥0.05 |
|  | p≥0.05 | **OLS ITS**  **Fixed MA** | | 0  vs  0 | 8  vs  9 | 0  vs  0 | 8  vs  9 | 0  vs  0 | 8  vs  9 | 0  vs  0 | 8  vs  9 | 1  vs  1 | 7  vs  8 | 0  vs  0 | 8  vs  9 | 0  vs  0 | 8  vs  9 | 0  vs  0 | 8  vs  9 | 0  vs  0 | 8  vs  9 |
|  | p<0.05 |  |  | 4  vs  4 | 5  vs  4 | 3  vs  3 | 6  vs  5 | 4  vs  4 | 5  vs  4 | 3  vs  3 | 6  vs  5 | 7  vs  7 | 2  vs  1 | 6  vs  5 | 3  vs  3 | 4  vs  3 | 5  vs  5 | 5  vs  5 | 4  vs  3 | 3  vs  3 | 6  vs  5 |
|  | p≥0.05 | 0  vs  0 | 7  vs  7 | **OLS ITS**  **DL+WT MA** | | 0  vs  0 | 13  vs  13 | 0  vs  0 | 13  vs  13 | 0  vs  0 | 13  vs  13 | 4  vs  4 | 9  vs  9 | 2  vs  1 | 11  vs  12 | 0  vs  0 | 13  vs  13 | 1  vs  1 | 12  vs  12 | 0  vs  0 | 13  vs  13 |
|  | p<0.05 | 6  vs  7 | 4  vs  3 |  |  | 3  vs  3 | 1  vs  1 | 4  vs  4 | 0  vs  0 | 3  vs  3 | 1  vs  1 | 4  vs  4 | 0  vs  0 | 4  vs  4 | 0  vs  0 | 4  vs  3 | 0  vs  1 | 4  vs  4 | 0  vs  0 | 3  vs  3 | 1  vs  1 |
|  | p≥0.05 | 0  vs  0 | 7  vs  7 | 0  vs  0 | 11  vs  10 | **OLS ITS**  **DL+HKSJ MA** | | 1  vs  1 | 13  vs  13 | 0  vs  0 | 14  vs  14 | 5  vs  5 | 9  vs  9 | 3  vs  2 | 11  vs  12 | 1  vs  1 | 13  vs  13 | 2  vs  2 | 12  vs  12 | 1  vs  1 | 13  vs  13 |
|  | p<0.05 | 4  vs  5 | 6  vs  5 | 4  vs  5 | 2  vs  2 |  |  | 3  vs  3 | 0  vs  0 | 3  vs  3 | 0  vs  0 | 3  vs  3 | 0  vs  0 | 3  vs  3 | 0  vs  0 | 3  vs  2 | 0  vs  1 | 3  vs  3 | 0  vs  0 | 2  vs  2 | 1  vs  1 |
|  | p≥0.05 | 0  vs  0 | 7  vs  7 | 0  vs  0 | 11  vs  10 | 1  vs  1 | 12  vs  11 | **OLS ITS**  **REML+WT MA** | | 0  vs  0 | 13  vs  13 | 4  vs  4 | 9  vs  9 | 2  vs  1 | 11  vs  12 | 0  vs  0 | 13  vs  13 | 1  vs  1 | 12  vs  12 | 0  vs  0 | 13  vs  13 |
|  | p<0.05 | 4  vs  5 | 6  vs  5 | 4  vs  5 | 2  vs  2 | 3  vs  4 | 1  vs  1 |  |  | 3  vs  3 | 1  vs  1 | 4  vs  4 | 0  vs  0 | 4  vs  4 | 0  vs  0 | 4  vs  3 | 0  vs  1 | 4  vs  4 | 0  vs  0 | 3  vs  3 | 1  vs  1 |
|  | p≥0.05 | 0  vs  0 | 7  vs  7 | 0  vs  0 | 11  vs  10 | 0  vs  0 | 13  vs  12 | 0  vs  0 | 13  vs  12 | **OLS ITS**  **REML+HKSJ MA** | | 5  vs  5 | 9  vs  9 | 3  vs  2 | 11  vs  12 | 1  vs  1 | 13  vs  13 | 2  vs  2 | 12  vs  12 | 1  vs  1 | 13  vs  13 |
|  | p<0.05 | 3  vs  4 | 7  vs  6 | 3  vs  4 | 3  vs  3 | 3  vs  4 | 1  vs  1 | 3  vs  4 | 1  vs  1 |  |  | 3  vs  3 | 0  vs  0 | 3  vs  3 | 0  vs  0 | 3  vs  2 | 0  vs  1 | 3  vs  3 | 0  vs  0 | 2  vs  2 | 1  vs  1 |
|  | p≥0.05 | 0  vs  0 | 7  vs  7 | 2  vs  1 | 9  vs  9 | 4  vs  3 | 9  vs  9 | 4  vs  3 | 9  vs  9 | 5  vs  4 | 9  vs  9 | **REML ITS**  **Fixed MA** | | 1  vs  0 | 8  vs  9 | 0  vs  0 | 9  vs  9 | 0  vs  0 | 9  vs  9 | 0  vs  0 | 9  vs  9 |
|  | p<0.05 | 7  vs  7 | 3  vs  3 | 5  vs  6 | 1  vs  1 | 3  vs  4 | 1  vs  1 | 3  vs  4 | 1  vs  1 | 2  vs  3 | 1  vs  1 |  |  | 5  vs  5 | 3  vs  3 | 4  vs  3 | 4  vs  5 | 5  vs  5 | 3  vs  3 | 3  vs  3 | 5  vs  5 |
|  | p≥0.05 | 0  vs  0 | 7  vs  7 | 0  vs  0 | 11  vs  10 | 1  vs  1 | 12  vs  11 | 1  vs  1 | 12  vs  11 | 2  vs  2 | 12  vs  11 | 0  vs  0 | 10  vs  10 | **REML ITS**  **DL+WT MA** | | 0  vs  0 | 11  vs  12 | 0  vs  0 | 11  vs  12 | 0  vs  0 | 11  vs  12 |
|  | p<0.05 | 4  vs  5 | 6  vs  5 | 4  vs  5 | 2  vs  2 | 3  vs  4 | 1  vs  1 | 3  vs  4 | 1  vs  1 | 2  vs  3 | 1  vs  1 | 4  vs  5 | 3  vs  2 |  |  | 4  vs  3 | 2  vs  2 | 5  vs  5 | 1  vs  0 | 3  vs  3 | 3  vs  2 |
|  | p≥0.05 | 0  vs  0 | 7  vs  7 | 0  vs  0 | 11  vs  10 | 0  vs  0 | 13  vs  12 | 0  vs  0 | 13  vs  12 | 0  vs  0 | 14  vs  13 | 1  vs  1 | 9  vs  9 | 1  vs  1 | 12  vs  11 | **REML ITS**  **DL+HKSJ MA** | | 1  vs  2 | 12  vs  12 | 0  vs  0 | 13  vs  14 |
|  | p<0.05 | 3  vs  4 | 7  vs  6 | 3  vs  4 | 3  vs  3 | 3  vs  4 | 1  vs  1 | 3  vs  4 | 1  vs  1 | 3  vs  4 | 0  vs  0 | 2  vs  3 | 5  vs  4 | 2  vs  3 | 2  vs  2 |  |  | 4  vs  3 | 0  vs  0 | 3  vs  3 | 1  vs  0 |
|  | p≥0.05 | 0  vs  0 | 7  vs  7 | 0  vs  0 | 11  vs  10 | 1  vs  1 | 12  vs  11 | 0  vs  0 | 13  vs  12 | 1  vs  1 | 13  vs  12 | 0  vs  0 | 10  vs  10 | 0  vs  0 | 13  vs  12 | 1  vs  1 | 13  vs  12 | **REML ITS**  **REML+WT MA** | | 0  vs  0 | 12  vs  12 |
|  | p<0.05 | 3  vs  4 | 7  vs  6 | 3  vs  4 | 3  vs  3 | 2  vs  3 | 2  vs  2 | 3  vs  4 | 1  vs  1 | 2  vs  3 | 1  vs  1 | 3  vs  4 | 4  vs  3 | 3  vs  4 | 1  vs  1 | 2  vs  3 | 1  vs  1 |  |  | 3  vs  3 | 2  vs  2 |
|  | p≥0.05 | 0  vs  0 | 7  vs  7 | 0  vs  0 | 11  vs  10 | 0  vs  0 | 13  vs  12 | 0  vs  0 | 13  vs  12 | 0  vs  0 | 14  vs  13 | 1  vs  1 | 9  vs  9 | 1  vs  1 | 12  vs  11 | 0  vs  0 | 14  vs  13 | 1  vs  1 | 13  vs  12 | **REML ITS**  **REML+HKSJ MA** | |
|  | p<0.05 | 3  vs  4 | 7  vs  6 | 3  vs  4 | 3  vs  3 | 3  vs  4 | 1  vs  1 | 3  vs  4 | 1  vs  1 | 3  vs  4 | 0  vs  0 | 2  vs  3 | 5  vs  4 | 2  vs  3 | 2  vs  2 | 3  vs  4 | 0  vs  0 | 2  vs  3 | 1  vs  1 |  |  |
| The top row of the label indicates the ITS analysis methods, bottom row indicates the meta-analysis method, e.g., OLS ITS Fixed MA indicates OLS ITS analysis and fixed effect meta-analysis was used. DL, DerSimonian and Laird. HKSJ, Hartung-Knapp / Sidik-Jonkman. ITS, interrupted time series. MA, meta-analysis. OLS, ordinary least squares. REML, restricted maximum likelihood. WT, Wald-type. | | | | | | | | | | | | | | | | | | | | | |

###

### Appendix Table S7. Comparing the primary and sensitivity analysis results for the percentage agreement (kappa statistic) in statistical significance of estimates of level-change and slope-change per month (categorised as p$\leq$0.05 or p>0.05) (n = 17)

|  | **Level-change** | | | | | | | | | |
| --- | --- | --- | --- | --- | --- | --- | --- | --- | --- | --- |
| **Slope-change per month** | **OLS ITS**  **Fixed MA** | 70.6% (0.43)  vs  76.5% (0.51) | 64.7% (0.32)  vs  70.6% (0.39) | 70.6% (0.43)  vs  76.5% (0.51) | 64.7% (0.32)  vs  70.6% (0.39) | 82.4% (0.65)  vs  88.2% (0.76) | 82.4% (0.65)  vs  82.4% (0.64) | 70.6% (0.43)  vs  70.6% (0.39) | 76.5% (0.54)  vs  82.4% (0.64) | 64.7% (0.32)  vs  70.6% (0.39) |
|  | 76.5% (0.55)  vs  82.4% (0.66) | **OLS ITS**  **DL+WT MA** | 94.1% (0.82)  vs  94.1% (0.82) | 100.0% (1.00)  vs  100.0% (1.00) | 94.1% (0.82)  vs  94.1% (0.82) | 76.5% (0.51)  vs  76.5% (0.51) | 88.2% (0.72)  vs  94.1% (0.85) | 100.0% (1.00)  vs  94.1% (0.82) | 94.1% (0.85)  vs  94.1% (0.85) | 94.1% (0.82)  vs  94.1% (0.82) |
|  | 64.7% (0.35)  vs  70.6% (0.45) | 88.2% (0.72)  vs  88.2% (0.75) | **OLS ITS**  **DL+HKSJ MA** | 94.1% (0.82)  vs  94.1% (0.82) | 100.0% (1.00)  vs  100.0% (1.00) | 70.6% (0.39)  vs  70.6% (0.39) | 82.4% (0.56)  vs  88.2% (0.68) | 94.1% (0.82)  vs  88.2% (0.60) | 88.2% (0.68)  vs  88.2% (0.68) | 88.2% (0.60)  vs  88.2% (0.60) |
|  | 64.7% (0.35)  vs  70.6% (0.45) | 88.2% (0.72)  vs  88.2% (0.75) | 88.2% (0.67)  vs  88.2% (0.72) | **OLS ITS**  **REML+WT MA** | 94.1% (0.82)  vs  94.1% (0.82) | 76.5% (0.51)  vs  76.5% (0.51) | 88.2% (0.72)  vs  94.1% (0.85) | 100.0% (1.00)  vs  94.1% (0.82) | 94.1% (0.85)  vs  94.1% (0.85) | 94.1% (0.82)  vs  94.1% (0.82) |
|  | 58.8% (0.26)  vs  64.7% (0.35) | 82.4% (0.56)  vs  82.4% (0.61) | 94.1% (0.82)  vs  94.1% (0.85) | 94.1% (0.82)  vs  94.1% (0.85) | **OLS ITS**  **REML+HKSJ MA** | 70.6% (0.39)  vs  70.6% (0.39) | 82.4% (0.56)  vs  88.2% (0.68) | 94.1% (0.82)  vs  88.2% (0.60) | 88.2% (0.68)  vs  88.2% (0.68) | 88.2% (0.60)  vs  88.2% (0.60) |
|  | 82.4% (0.66)  vs  82.4% (0.66) | 82.4% (0.63)  vs  88.2% (0.76) | 70.6% (0.35)  vs  76.5% (0.49) | 70.6% (0.35)  vs  76.5% (0.49) | 64.7% (0.20)  vs  70.6% (0.35) | **REML ITS**  **Fixed MA** | 76.5% (0.52)  vs  82.4% (0.64) | 76.5% (0.51)  vs  70.6% (0.39) | 82.4% (0.64)  vs  82.4% (0.64) | 70.6% (0.39)  vs  70.6% (0.39) |
|  | 64.7% (0.35)  vs  70.6% (0.45) | 88.2% (0.72)  vs  88.2% (0.75) | 88.2% (0.67)  vs  88.2% (0.72) | 88.2% (0.67)  vs  88.2% (0.72) | 82.4% (0.46)  vs  82.4% (0.55) | 82.4% (0.61)  vs  88.2% (0.75) | **REML ITS**  **DL+WT MA** | 88.2% (0.72)  vs  88.2% (0.68) | 94.1% (0.87)  vs  100.0% (1.00) | 82.4% (0.56)  vs  88.2% (0.68) |
|  | 58.8% (0.26)  vs  64.7% (0.35) | 82.4% (0.56)  vs  82.4% (0.61) | 94.1% (0.82)  vs  94.1% (0.85) | 94.1% (0.82)  vs  94.1% (0.85) | 100.0% (1.00)  vs  100.0% (1.00) | 64.7% (0.20)  vs  70.6% (0.35) | 82.4% (0.46)  vs  82.4% (0.55) | **REML ITS**  **DL+HKSJ MA** | 94.1% (0.85)  vs  88.2% (0.68) | 94.1% (0.82)  vs  100.0% (1.00) |
|  | 58.8% (0.26)  vs  64.7% (0.35) | 82.4% (0.56)  vs  82.4% (0.61) | 82.4% (0.46)  vs  82.4% (0.55) | 94.1% (0.82)  vs  94.1% (0.85) | 88.2% (0.60)  vs  88.2% (0.67) | 76.5% (0.47)  vs  82.4% (0.61) | 94.1% (0.82)  vs  94.1% (0.85) | 88.2% (0.60)  vs  88.2% (0.67) | **REML ITS**  **REML+WT MA** | 88.2% (0.68)  vs  88.2% (0.68) |
|  | 58.8% (0.26)  vs  64.7% (0.35) | 82.4% (0.56)  vs  82.4% (0.61) | 94.1% (0.82)  vs  94.1% (0.85) | 94.1% (0.82)  vs  94.1% (0.85) | 100.0% (1.00)  vs  100.0% (1.00) | 64.7% (0.20)  vs  70.6% (0.35) | 82.4% (0.46)  vs  82.4% (0.55) | 100.0% (1.00)  vs  100.0% (1.00) | 88.2% (0.60)  vs  88.2% (0.67) | **REML ITS**  **REML+HKSJ MA** |
| The top row of the label indicates the ITS analysis methods, bottom row indicates the meta-analysis method, e.g., OLS ITS Fixed MA is OLS ITS analysis and fixed effect meta-analysis. We used the following adjectives to describe agreement: moderate agreement as a kappa value of 0.41-0.6, substantial agreement as a value of 0.61-0.8, and almost perfect agreement as a value of 0.81-1.0.  DL, DerSimonian and Laird. HKSJ, Hartung-Knapp / Sidik-Jonkman. ITS, interrupted time series. MA, meta-analysis. OLS, ordinary least squares. REML, restricted maximum likelihood. WT, Wald-type. | | | | | | | | | | |

###

### Appendix Table S8. Comparing the primary and sensitivity analysis results for the median and IQR for the differences in between-study variance estimates for the meta-analytic immediate level-change (top triangle, difference calculated as column method - row method) and slope-change per month (bottom triangle, difference calculated as row method - column method).

|  | **Level-change** | | | |
| --- | --- | --- | --- | --- |
| **Slope-change per month** | **OLS ITS**  **DL MA** | 0.00 (0.00,0.38)  vs  0.00 (0.00,0.03) | 0.00 (-0.28,0.00)  vs  0.00 (-0.28,0.00) | 0.00 (0.00,0.83)  vs  0.00 (-0.01,1.29) |
|  | 0.00 (0.00,0.00)  vs  0.00 (0.00,0.00) | **OLS ITS**  **REML MA** | 0.00 (-0.29,0.00)  vs  0.00 (-0.29,0.00) | 0.00 (-0.29,0.08)  vs  0.00 (-0.26,0.08) |
|  | 0.00 (0.00,0.01)  vs  0.00 (0.00,0.01) | 0.00 (-0.01,0.00)  vs  0.00 (0.00,0.00) | **REML ITS**  **DL MA** | 0.00 (0.00,0.08)  vs  0.00 (0.00,0.08) |
|  | 0.00 (0.00,0.03)  vs  0.00 (0.00,0.07) | 0.00 (0.00,0.02)  vs  0.00 (0.00,0.03) | 0.00 (0.00,0.00)  vs  0.00 (0.00,0.03) | **REML ITS**  **REML MA** |
| For example, the median difference in between-study variance for immediate level-change yielded by REML ITS analysis with DL estimator and OLS ITS analysis with DL estimator was 0.00 for both the primary and sensitivity analyses (column 3, row 1). The same was observed for slope-change per month (column 1, row 3).  DL, DerSimonian and Laird. HKSJ, Hartung-Knapp / Sidik-Jonkman. ITS, interrupted time series. IQR, interquartile range. MA, meta-analysis. OLS, ordinary least squares. REML, restricted maximum likelihood. WT, Wald-type. | | | | |

## Appendix 4 – Reviews that contributed data

The table below contains the citations of the 40 reviews that met our eligibility criteria. We indicate whether and how data was provided for each review and whether the data has been made available in our online repository. We thank all authors who contributed data for this study.

### Appendix Table S9. Eligible reviews and reviews that contributed data

| **Citation** | **Data source** | **Data is supplied in online repository** |
| --- | --- | --- |
| Acosta A, Ciapponi A, Aaserud M, Vietto V, Austvoll-Dahlgren A, Kösters JP, Vacca C, Machado M, Diaz Ayala DH, Oxman AD. Pharmaceutical policies: effects of reference pricing, other pricing, and purchasing policies. Cochrane Database Syst Rev. 2014 Oct 16;2014(10):CD005979. doi: 10.1002/14651858.CD005979.pub2. PMID: 25318966; PMCID: PMC6703418. | Not provided | No |
| Baccini M, Carreras G. Analyzing and comparing the association between control policy measures and alcohol consumption in Europe. Subst Use Misuse. 2014 Oct;49(12):1684-91. doi: 10.3109/10826084.2014.914373. Epub 2014 May 15. PMID: 24832722. | Not provided | No |
| Blot K, Bergs J, Vogelaers D, Blot S, Vandijck D. Prevention of central line-associated bloodstream infections through quality improvement interventions: a systematic review and meta-analysis. Clin Infect Dis. 2014 Jul 1;59(1):96-105. doi: 10.1093/cid/ciu239. Epub 2014 Apr 9. PMID: 24723276; PMCID: PMC4305144. | Not provided | No |
| Chaillet N, Dumont A. Evidence-based strategies for reducing cesarean section rates: a meta-analysis. Birth. 2007 Mar;34(1):53-64. doi: 10.1111/j.1523-536X.2006.00146.x. PMID: 17324180. | Not provided | No |
| Crifasi CK, Pollack KM, Webster DW. Effects of state-level policy changes on homicide and nonfatal shootings of law enforcement officers. Inj Prev. 2016 Aug;22(4):274-8. doi: 10.1136/injuryprev-2015-041825. Epub 2015 Dec 30. PMID: 26718550. | Data provided by authors | Yes |
| Davey P, Marwick CA, Scott CL, Charani E, McNeil K, Brown E, Gould IM, Ramsay CR, Michie S. Interventions to improve antibiotic prescribing practices for hospital inpatients. Cochrane Database Syst Rev. 2017 Feb 9;2(2):CD003543. doi: 10.1002/14651858.CD003543.pub4. PMID: 28178770; PMCID: PMC6464541. | Data provided by authors | Yes |
| Ejlerskov KT, Sharp SJ, Stead M, Adamson AJ, White M, Adams J. Supermarket policies on less-healthy food at checkouts: Natural experimental evaluation using interrupted time series analyses of purchases. PLoS Med. 2018 Dec 18;15(12):e1002712. doi: 10.1371/journal.pmed.1002712. PMID: 30562349; PMCID: PMC6298641. | Data digitally extracted | Yes |
| Ferri M, Allara E, Bo A, Gasparrini A, Faggiano F. Media campaigns for the prevention of illicit drug use in young people. Cochrane Database Syst Rev. 2013 Jun 5;(6):CD009287. doi: 10.1002/14651858.CD009287.pub2. PMID: 23740538. | Not provided | No |
| Fu KW, Yip PS. Estimating the risk for suicide following the suicide deaths of 3 Asian entertainment celebrities: a meta-analytic approach. J Clin Psychiatry. 2009 Jun;70(6):869-78. doi: 10.4088/jcp.08m04240. PMID: 19573483. | Not provided | No |
| Garcia-Elorrio E, Rowe SY, Teijeiro ME, Ciapponi A, Rowe AK. The effectiveness of the quality improvement collaborative strategy in low- and middle-income countries: A systematic review and meta-analysis. PLoS One. 2019 Oct 3;14(10):e0221919. doi: 10.1371/journal.pone.0221919. PMID: 31581197; PMCID: PMC6776335. | Data provided by authors | Yes |
| Giguère A, Légaré F, Grimshaw J, Turcotte S, Fiander M, Grudniewicz A, Makosso-Kallyth S, Wolf FM, Farmer AP, Gagnon MP. Printed educational materials: effects on professional practice and healthcare outcomes. Cochrane Database Syst Rev. 2012 Oct 17;10(10):CD004398. doi: 10.1002/14651858.CD004398.pub3. Update in: Cochrane Database Syst Rev. 2020 Jul 31;8:CD004398. PMID: 23076904; PMCID: PMC7197046. | Data provided by authors | No |
| Goldzweig CL, Orshansky G, Paige NM, Miake-Lye IM, Beroes JM, Ewing BA, Shekelle PG. Electronic health record-based interventions for improving appropriate diagnostic imaging: a systematic review and meta-analysis. Ann Intern Med. 2015 Apr 21;162(8):557-65. doi: 10.7326/M14-2600. PMID: 25894025. | Not provided | No |
| Guignet D, Jenkins R, Ranson M, Walsh P. Contamination and Incomplete Information:: Bounding Implicit Prices using High-Profile Leaks. J Environ Econ Manage. 2018;88:259-282. doi: 10.1016/j.jeem.2017.12.003. PMID: 30996495; PMCID: PMC6463534. | Not provided | No |
| Harder T, Siedler A. Systematic Review and Meta-analysis of Chickenpox Vaccination and Risk of Herpes Zoster: A Quantitative View on the "Exogenous Boosting Hypothesis". Clin Infect Dis. 2019 Sep 27;69(8):1329-1338. doi: 10.1093/cid/ciy1099. PMID: 30590491. | Not provided | No |
| Kim Y, Gasparrini A, Hashizume M, Honda Y, Ng CFS, Armstrong B. Heat-Related Mortality in Japan after the 2011 Fukushima Disaster: An Analysis of Potential Influence of Reduced Electricity Consumption. Environ Health Perspect. 2017 Jul 6;125(7):077005. doi: 10.1289/EHP493. PMID: 28686555; PMCID: PMC5744700. | Not provided | No |
| Lane TJ, Hall W. Traffic fatalities within US states that have legalized recreational cannabis sales and their neighbours. Addiction. 2019 May;114(5):847-856. doi: 10.1111/add.14536. Epub 2019 Feb 4. PMID: 30719794. | Data provided in publication | Yes |
| Larance B, Dobbins T, Peacock A, Ali R, Bruno R, Lintzeris N, Farrell M, Degenhardt L. The effect of a potentially tamper-resistant oxycodone formulation on opioid use and harm: main findings of the National Opioid Medications Abuse Deterrence (NOMAD) study. Lancet Psychiatry. 2018 Feb;5(2):155-166. doi: 10.1016/S2215-0366(18)30003-8. Epub 2018 Jan 11. PMID: 29336948. | Not provided | No |
| Li Q, Wen M. The Immediate and Lingering Effects of Armed Conflict on Adult Mortality: A Time-Series Cross-National Analysis. Journal of Peace Research. 2005;42(4):471-492. doi:10.1177/0022343305054092. | Not provided | No |
| Luangasanatip N, Hongsuwan M, Limmathurotsakul D, Lubell Y, Lee AS, Harbarth S, Day NP, Graves N, Cooper BS. Comparative efficacy of interventions to promote hand hygiene in hospital: systematic review and network meta-analysis. BMJ. 2015 Jul 28;351:h3728. doi: 10.1136/bmj.h3728. PMID: 26220070; PMCID: PMC4517539. | Data digitally extracted | Yes |
| Perkins C, Steinbach R, Tompson L, Green J, Johnson S, Grundy C, Wilkinson P, Edwards P. What is the effect of reduced street lighting on crime and road traffic injuries at night? A mixed-methods study. Southampton (UK): NIHR Journals Library; 2015 Sep. PMID: 26401542. | Not provided | No |
| Phung D, Chu C, Rutherford S, Nguyen HLT, Luong MA, Do CM, Huang C. Heavy rainfall and risk of infectious intestinal diseases in the most populous city in Vietnam. Sci Total Environ. 2017 Feb 15;580:805-812. doi: 10.1016/j.scitotenv.2016.12.027. Epub 2016 Dec 21. PMID: 28012659. | Data provided by authors | Yes |
| Phung D, Nguyen HT, Chu C, Sadler R, Luong AM, Nguyen HT, Pham TC, Huang C. Impacts of helmet law on the changes in potential years of life lost due to traffic injury: a multiple-province evaluation in Vietnam. Inj Prev. 2020 Apr;26(2):109-115. doi: 10.1136/injuryprev-2018-043088. Epub 2019 Mar 5. PMID: 30837327. | Data provided by authors | Yes |
| Phung D, Tran PD, Nguyen LH, Do CM, Rutherford S, Chu C. The impact of prevention and control of infectious disease law on diarrhoea control: a 5-year evaluation in multiple provinces in Vietnam. Health Policy Plan. 2017 Dec 1;32(10):1347-1353. doi: 10.1093/heapol/czx099. PMID: 28973652. | Data provided by authors | Yes |
| Pryce J, Choi L, Richardson M, Malone D. Insecticide space spraying for preventing malaria transmission. Cochrane Database Syst Rev. 2018 Nov 2;11(11):CD012689. doi: 10.1002/14651858.CD012689.pub2. PMID: 30388303; PMCID: PMC6516806. | Data provided by authors | Yes |
| Rashidian A, Omidvari AH, Vali Y, Sturm H, Oxman AD. Pharmaceutical policies: effects of financial incentives for prescribers. Cochrane Database Syst Rev. 2015 Aug 4;2015(8):CD006731. doi: 10.1002/14651858.CD006731.pub2. PMID: 26239041; PMCID: PMC7390265. | Not provided | No |
| Rautiainen RH, Lehtola MM, Day LM, Schonstein E, Suutarinen J, Salminen S, Verbeek J. Interventions for preventing injuries in the agricultural industry. Cochrane Database Syst Rev. 2008 Jan 23;(1):CD006398. doi: 10.1002/14651858.CD006398.pub2. PMID: 18254102. | Data provided by authors | Yes |
| Reddy VK, Lavoie MC, Verbeek JH, Pahwa M. Devices for preventing percutaneous exposure injuries caused by needles in healthcare personnel. Cochrane Database Syst Rev. 2017 Nov 14;11(11):CD009740. doi: 10.1002/14651858.CD009740.pub3. PMID: 29190036; PMCID: PMC6491125. | Data provided by authors | Yes |
| Ripoll Gallardo A, Pacelli B, Alesina M, Serrone D, Iacutone G, Faggiano F, Della Corte F, Allara E. Medium- and long-term health effects of earthquakes in high-income countries: a systematic review and meta-analysis. Int J Epidemiol. 2018 Aug 1;47(4):1317-1332. doi: 10.1093/ije/dyy130. PMID: 30053061. | Not provided | No |
| Rowe AK, Rowe SY, Peters DH, Holloway KA, Chalker J, Ross-Degnan D. Effectiveness of strategies to improve health-care provider practices in low-income and middle-income countries: a systematic review. Lancet Glob Health. 2018 Nov;6(11):e1163-e1175. doi: 10.1016/S2214-109X(18)30398-X. Epub 2018 Oct 8. PMID: 30309799; PMCID: PMC6185992. | Data provided by authors | Yes |
| Tippetts AS, Voas RB, Fell JC, Nichols JL. A meta-analysis of .08 BAC laws in 19 jurisdictions in the United States. Accid Anal Prev. 2005 Jan;37(1):149-61. doi: 10.1016/j.aap.2004.02.006. PMID: 15607286. | Not provided | No |
| van der Molen HF, Basnet P, Hoonakker PL, Lehtola MM, Lappalainen J, Frings-Dresen MH, Haslam R, Verbeek JH. Interventions to prevent injuries in construction workers. Cochrane Database Syst Rev. 2018 Feb 5;2(2):CD006251. doi: 10.1002/14651858.CD006251.pub4. PMID: 29400395; PMCID: PMC6491133. | Data provided by authors | Yes |
| Vicedo-Cabrera AM, Röösli M, Radovanovic D, Grize L, Witassek F, Schindler C, Perez L. Cardiorespiratory hospitalisation and mortality reductions after smoking bans in Switzerland. Swiss Med Wkly. 2016 Dec 19;146:w14381. doi: 10.4414/smw.2016.14381. PMID: 28102874. | Not provided | No |
| Vicedo-Cabrera AM, Schindler C, Radovanovic D, Grize L, Witassek F, Dratva J, Röösli M, Perez L. Benefits of smoking bans on preterm and early-term births: a natural experimental design in Switzerland. Tob Control. 2016 Dec;25(e2):e135-e141. doi: 10.1136/tobaccocontrol-2015-052739. Epub 2016 Apr 26. PMID: 27118814. | Not provided | No |
| Vidanapathirana J, Abramson MJ, Forbes A, Fairley C. Mass media interventions for promoting HIV testing. Cochrane Database Syst Rev. 2005 Jul 20;(3):CD004775. doi: 10.1002/14651858.CD004775.pub2. PMID: 16034948. | Data provided by authors | Yes |
| Wagenaar AC, Maldonado-Molina MM, Erickson DJ, Ma L, Tobler AL, Komro KA. General deterrence effects of U.S. statutory DUI fine and jail penalties: long-term follow-up in 32 states. Accid Anal Prev. 2007 Sep;39(5):982-94. doi: 10.1016/j.aap.2007.01.003. Epub 2007 Mar 21. PMID: 17854574. | Not provided | No |
| Wagenaar AC, Maldonado-Molina MM, Ma L, Tobler AL, Komro KA. Effects of legal BAC limits on fatal crash involvement: analyses of 28 states from 1976 through 2002. J Safety Res. 2007;38(5):493-9. doi: 10.1016/j.jsr.2007.06.001. Epub 2007 Sep 29. PMID: 18023634. | Not provided | No |
| Weatherburn CJ, Guthrie B, Dreischulte T, Morales DR. Impact of medicines regulatory risk communications in the UK on prescribing and clinical outcomes: Systematic review, time series analysis and meta-analysis. Br J Clin Pharmacol. 2020 Apr;86(4):698-710. doi: 10.1111/bcp.14104. Epub 2019 Dec 16. Erratum in: Br J Clin Pharmacol. 2020 Sep;86(9):1894. PMID: 31465123; PMCID: PMC7098864. | Not provided | No |
| Wilson DB, Olaghere A and Gill C. Juvenile curfew effects on criminal behavior and victimization: A Campbell Collaboration systematic review. Journal of Experimental Criminology 2016; 12: 167-186. Literature Review; Systematic Review. DOI: <http://dx.doi.org/10.1007/s11292-016-9258-y>. | Data provided in publication | Yes |
| Yinon L, Thurston G. An evaluation of the health benefits achieved at the time of an air quality intervention in three Israeli cities. Environ Int. 2017 May;102:66-73. doi: 10.1016/j.envint.2016.12.025. Epub 2017 Feb 23. PMID: 28237065; PMCID: PMC5771478. | Not provided | No |
| Zhang N, Song D, Zhang J, Liao W, Miao K, Zhong S, Lin S, Hajat S, Yang L, Huang C. The impact of the 2016 flood event in Anhui Province, China on infectious diarrhea disease: An interrupted time-series study. Environ Int. 2019 Jun;127:801-809. doi: 10.1016/j.envint.2019.03.063. Epub 2019 Apr 30. PMID: 31051323. | Not provided | No |

## References

1. Ejlerskov KT, Sharp SJ, Stead M, et al. Supermarket policies on less-healthy food at checkouts: Natural experimental evaluation using interrupted time series analyses of purchases. *PLOS Med* 2018; 15: e1002712. Research Support, Non-U.S. Gov't. DOI: https://doi.org/10.1371/journal.pmed.1002712.

2. Phung D, Chu C, Rutherford S, et al. Heavy rainfall and risk of infectious intestinal diseases in the most populous city in Vietnam. *Sci Total Environ* 2017; 580: 805-812. DOI: https://doi.org/10.1016/j.scitotenv.2016.12.027.
